# Human pancreatic cancer patients with Epithelial-to-Mesenchymal Transition and an aggressive phenotype show a disturbed balance in Protein Phosphatase Type 2A expression and functionality

**DOI:** 10.1186/s12967-023-04145-z

**Published:** 2023-05-11

**Authors:** Jos van Pelt, Bob Meeusen, Rita Derua, Liesbeth Guffens, Eric Van Cutsem, Veerle Janssens, Chris Verslype

**Affiliations:** 1 Laboratory of Digestive Oncology, Department of Oncology, KU Leuven & University Hospitals Leuven, Geb. Onderwijs & Navorsing 4, Room 07.465, Herestraat 49, Bus 603, B3000 Leuven, Belgium; 2 KU Leuven Cancer Institute (LKI), Herestraat 49, B3000 Leuven, Belgium; 3 Laboratory of Protein Phosphorylation & Proteomics, Dept. of Cellular & Molecular Medicine, O&N1, University of Leuven (KU Leuven), Herestraat 49, Bus 901, B3000 Leuven, Belgium; 4 SyBioMa (KU Leuven), Herestraat 49, B3000 Leuven, Belgium

**Keywords:** Pancreatic cancer, Epithelial-to-mesenchymal progression, Protein phosphatase type 2A, Gene expression, Proteomics, In vivo results, Drug target

## Abstract

**Background:**

Pancreatic ductal adenocarcinoma (PDAC) has a low survival, its incidence is rising and little therapeutic improvements are expected in the near future. It has been observed that Epithelial-to-Mesenchymal transition (EMT) contributes (including in PDAC) to a more aggressive cancer phenotype. Additionally, largely unexplored, studies indicate a mechanistic interplay between Protein Phosphatase Type 2A (PP2A) enzymes and EMT that could offer treatment opportunities. The aim was to investigate the relation of a PP2A expression signature (encompassing all PP2A subunits, endogenous inhibitors and activators) with EMT and aggressive pancreatic cancer, and to discuss possible implications.

**Methods:**

We retrieved different PDAC expression datasets from NCBI to capture the variation in patients, and analyzed these using datamining, survival analysis, differential gene and protein expression. We determined genes highly associated with aggressive PDAC. For in vitro evaluation, Panc-1 cells were treated with the pharmacologic PP2A inhibitor Okadaic Acid (OA). Additionally, two OA-resistant Panc-1 clones were developed and characterized.

**Results:**

In patients, there is a strong correlation between EMT and aggressive PDAC, and between aggressive PDAC and PP2A, with a significant upregulation of PP2A inhibitor genes. Several PP2A genes significantly correlated with decreased survival. In vitro, short-term exposure to OA induced EMT in Panc-1 cells. This shift towards EMT was further pronounced in the OA-resistant Panc-1 clones, morphologically and by pathway analysis. Proteomic analysis and gene sequencing showed that the advanced OA-resistant model most resembles the clinical PDAC presentation (with EMT signature, and with several specific PP2A genes upregulated, and others downregulated).

**Conclusions:**

We demonstrated a strong association between EMT, altered PP2A expression and aggressive PDAC in patients. Also, in vitro, PP2A inhibition induces EMT. Overall, statistics suggests the mechanistic importance of PP2A dysregulation for PDAC progression. Translationally, our observations indicate that pharmacologic restoration of PP2A activity could be an attractive therapeutic strategy to block or reverse progression.

**Supplementary Information:**

The online version contains supplementary material available at 10.1186/s12967-023-04145-z.

## Background

Pancreatic ductal adenocarcinoma (PDAC) is the most common type of pancreatic cancer (> 90%). Its incidence is rising, and it has a low 5-year survival rate (7–8%) [1]. Treatment approaches for PDAC are surgery, radiation therapy and chemotherapy, which may prolong survival and/or relieve symptoms for the patient but seldom result in a real cure. Patients with localized disease are treated with integrated therapy based on radical surgery; still there is little improvement to be expected in the near future [2]. Several chemotherapeutic drugs are being used in the clinic, but most patients do not respond well and end up with resistance and disease progression [2]. Other targeted therapies are also under pre-clinical investigation, but none have been accepted for general use [3] and also immunotherapy trials have shown no clinical benefit [4].

Not all PDAC are the same and it is now clear that there exists considerable intra-tumoral heterogeneity [5–9], which poses a major challenge to personalized treatments. In recent years, several studies have been performed to determine pancreatic subtypes based on gene expression [5, 6], genomics [7], proteomics [8] or immunotyping [9], resulting in between two and six subtypes. The approach of unsupervised molecular cancer classification aims to identify a tumor, or a sub-class of tumors based on markers that are highly discriminatory, while these markers do not have to have an apparent relationship per se. This classification can then be used in the therapeutic process to improve treatment. Alternatively, as was used in this study, a more mechanistic approach can be used for molecular classification and targeted intervention.

Pancreatic tumors are unique with a high stromal content (up to 90%) and the cancer–stroma interplay is important for tumor progression and drug delivery [10]. Various biochemical and biophysical factors in the tumor microenvironment, including hypoxia [11], TGF-β1, growth factors and cytokines are capable of inducing Epithelial-to-Mesenchymal Transition (EMT). EMT represents a collection of functional changes to the cells and is considered a driver for drug-resistance, migratory capacity, invasion, development of fibrosis, metabolic changes and cancer progression [12]. The hallmark EMT gene set includes more than 250 genes [13, 14] and depending on which triggers are active, the cell develops a number of these characteristics, as EMT is dynamic and reversible. During the process of EMT, the cells undergo morphological changes, lose epithelial markers such as E-cadherin and certain cytokeratins, and gain mesenchymal markers such as N-cadherin and vimentin; furthermore, they show increased proliferation [15]. Overall, the cells become less dependent on cell–cell contacts for their growth, resulting in increased invasion and metastasis. These changes are mediated by transcription factors, including *SNAI1*, *SNAI2*, *TWIST* and *ZEB*. In PDAC patients, an EMT-high phenotype is associated with tumor proliferation and shorter survival [16].

The second mechanism in pancreatic cancer that we want to examine involves protein phosphatases of type 2A (PP2A). This is a group of closely related enzymes that are central to several cellular processes especially control of cell survival and proliferation (Additional file 1: Fig. S1A) [17–19]. PP2A phosphatases are widely regarded as tumor suppressors but these enzymes have so far largely been overlooked in the context of clinical oncology or as therapeutic target [20]. Structurally, PP2A phosphatases are composed of at least two subunits, one of which is the catalytic C subunit, and one is the structural A subunit (Additional file 1: Fig. S1B) [21]. In the majority of cases, however, PP2A phosphatases are heterotrimeric enzymes, formed by the association of the A-C core dimer with a regulatory B-type subunit [21]. In human cells, these three subunit classes are encoded by 19 different genes (Additional file 1: Fig. S1B). The combinatorial assembly of one subunit from each class gives rise to multiple PP2A holoenzymes within a tissue, each with a defined substrate specificity and activity [21]. PP2A activity is further regulated by several endogenous inhibitors (e.g., KIAA1524/CIP2A, SET), the expression of which is often increased in cancer cells [22, 23], and by cellular activators (e.g., PTPA), the expression of which is often decreased in tumors [24, 25] (Additional file 1: Fig. S1B).

Several reports (predominantly from in vitro studies) have found the involvement of individual PP2A genes or their inhibitors in EMT and aggressive forms of cancer [reviewed in 20, 23, 26]. The B55α subunit of PP2A (encoded by *PPP2R2A*) showed higher expression in PDAC compared to adjacent normal tissue. In mouse models, B55α supports the tumorigenic and metastatic potential of pancreatic cancer cells [27]. In PDAC, activated YAP1 has been associated with EMT plasticity and metastasis induction [28] in which B55α may play a role [29]. Overexpression of CIP2A in PDAC correlated with EMT gene expression changes [30] and was associated with poor prognosis. Mody et al. found a correlation of SET isoform 2 overexpression with the EMT-marker N-cadherin in pancreatic cancer in vitro [31]. Li et al. found that downregulation of SETBP1 in non-small cell lung cancer induces EMT and cancer progression [32]. The pharmacologic inhibitor of PPP-like serine/threonine protein phosphatases, Okadaic acid (OA), has been identified as an EMT and tumor promoter in hepatocellular cellular carcinoma in vitro by a mechanism involving inhibition of PP2A [33].

In the present study, we specifically aimed to investigate the relation of the spectrum of PP2A-related genes (catalytic, structural and regulatory subunits and the major PP2A regulators and inhibitors) with mesenchymal gene expression in PDAC patients. This should give us insight into the modulation of gene expression under influence of the microenvironment in the context of natural cancer development (effect of time and adaptation) and cancer progression. Furthermore, we explored the PP2A-genes as tumor markers. The effect of short-term and long-term deregulation of PP2A by OA in relation to EMT was further studied in vitro using the human Panc-1 PDAC cell line. These questions on putative PP2A-EMT interrelations in PDAC have currently become more important with the identification of PP2A targeting compounds (e.g., LB100, OA, Cantharidin) [34–36] and the recent development of small molecules that can stimulate (inhibited) PP2A activity (e.g. FTY720, “SMAP (small-molecule activators of PP2A)”) [18, 37–40] in relation to cancer treatment.

## Methods

### Patient datasets and characteristics

We investigated gene expression in five patient datasets; one dataset was from our own institute (GSE62165) and four were externally available through NCBI (GSE15471, GSE16515, GSE17891 and GSE101448). Datasets GSE15471 and GSE17891 were used by Collisson et al. to determine the PDAC molecular classification [5]. Three datasets include information of tumor versus non-tumor (GSE15471, GSE16515 and GSE101448) and GSE62165 provides information on EMT-high versus EMT-low PDAC. All the GSE datasets used in this paper are publicly accessible through the NCBI website (https://www.ncbi.nlm.nih.gov/). Clinical evaluation was further done for gene expression in the TCGA dataset, and for protein expression we used the data from CPTAC Confirmatory/Discovery dataset [41].

### Molecular classification

#### Pancreatic cancer

Collisson et al. performed microarray on 27 micro-dissected samples and analyzed them in combination with expression data from a number of pancreatic cell lines and expression results from the Baeda dataset [42]. They identified a set of 62 genes that could classify the PDAC into subclasses (PDAssign, Additional file 1: Table S1) [5]. The performance of these sixty-two genes was confirmed in the study by Janky et al. [43].

#### Epithelial-to-mesenchymal transition

For hierarchical clustering and association studies we used the genes that are linked with EMT by the IPA-consortium. This web-based software is backed by the Ingenuity Knowledge Base of highly structured, detail-rich biological and chemical findings obtained through datamining in literature and other databases, and is constantly updated (Additional file 1: Table S1). The IPA-EMT gene list is focused; it contains less and some other genes than the MSigDB gene list from GSEA. Both IPA and MSigDB are not specific for one single type of tissue or only for cancer but describe EMT in general markers.

Protein phosphatase 2 (PP2A): We selected a list of 59 search terms (gene symbols and synonyms) based on literature (Additional file 1: Table S1).

#### Gene set enrichment analysis (GSEA)

Unsupervised analysis was performed using GSEA software version 4.1.0 (release July 2020) and Molecular Signature Database (MSigDB) 7.4 hallmark gene sets [release April 2021, 13].

### PP2A inhibition, viability and EMT in human PDAC cells

Human PDAC Panc-1 cells were obtained from ATCC (cat nr. CRL-1469, Gaithersburg, MD 20877, USA). Unless otherwise indicated serum, media and supplements used for cell culture experiments were obtained from Thermo Fischer Scientific, Waltham, Massachusetts, USA. Cells were cultured in DMEM medium supplemented with 10% Fetal bovine serum (FBS), 2 mM l-glutamine, 100 U/ml Penicillin and 100 µg/ml Streptomycin at 37 °C and 5% CO_2_. In vitro, the involvement of PP2A with EMT-induction in Panc-1 cells was studied following acute exposure of serum-starved cells (grown overnight in DMEM + 1% FBS) to 20 nM of the PP2A inhibitor Okadaic acid (OA, Calbiochem, USA) for 24 h, 48 h or 72 h. Percentage cell viability was measured by staining trypsinized cells (including detached cells) with trypan blue (T8154, Sigma), followed by cell counting with the automated cell counter Luna-II (Westburg). Protein lysates were prepared in RIPA buffer, supplemented with protease and phosphatase inhibitor cocktail tablets (Roche Applied Science). Protein concentration of the lysates was measured by the BCA method, and samples of equal concentration were prepared in SDS-PAGE sample buffer for subsequent analysis by Western blotting.

We also developed a Panc-1 cell model that survived in the presence of OA above its IC50, using the approach we previously described for sorafenib-resistance in HepG2 cells [44]. In short, Panc-1 cells were cultured in 6-well plate in DMEM medium supplemented with 10% FBS, 2 mM l-glutamine, 100 U/ml Penicillin and 100 µg/ml Streptomycin in the presence of 2 nM OA for 3 passages (below IC50). After the third passage the surviving cells were reseeded in medium with incremental step of 0.5 nM OA and the process repeated. When the cells in culture survived and multiplied at 8 nM OA, they were detached and seeded in a 96-well plate in serial dilution of 100 to 0.3 cells/well for clone selection. Eight wells that had outgrowth of a single colony were selected and separately expanded and characterized at the morphological (phase-contrast microscopy), molecular and proteomics level which resulted in the clones described in this study. For naïve Panc-1 cells, a dose of ≥ 8 nM for > 72 h induces their inability to multiply, followed by cell death.

### Molecular and biochemical techniques used for cell line characterization

#### RNA sequencing

RNA was isolated with the RNeasy Kit (Qiagen, Chatsworth, CA) according to the manufacturer's instructions. RNA sequencing and processing were performed by the VIB Nucleomics Core (www.nucleomics.be). In short, the poly-A containing mRNA molecules were purified, converted into cDNA and sequence-libraries of each sample were equimolarly pooled and sequenced on Illumina NovaSeq 6000 (100 bp, Single Reads, v1.5) (for full methods: see Additional file 1). RNA sequencing data were deposited at Gene Expression Omnibus (NCBI: GSE216036).

#### Western blot

Ten to twenty micrograms of protein per condition were separated on a NuPage 4–12% Bis–Tris gel (WG1401, Thermo Fischer Scientific, Waltham, Massachusetts, USA). Proteins were transferred to nitrocellulose membrane by wet blotting. Membranes were blocked in 5% milk in TBS/0.1% Tween-20 for 1 h at room temperature and incubated with the primary antibody overnight at 4 °C. Primary antibodies were: mouse monoclonal anti-vinculin (Sigma, V9131) and anti-PP2A-C (in-house, gift from Dr. S. Dilworth, London, UK); rabbit anti-Snail (Cell Signaling Technologies, 3879T) and anti-Epcam (Abcam, AB71916). After washing in TBS/0.1% Tween-20, membranes were incubated with horseradish peroxidase–conjugated secondary antibodies (Dako for anti-mouse and Cell Signaling for anti-rabbit) for 1 h and developed using Western Bright ECL (Advansta) on the ImageQuant LAS4000 scanner (GE Healthcare). Primary antibodies were used in a dilution of 1/1000, the secondary antibodies were used in a dilution of 1/5000. All densitometric quantifications were done with Image Studio Lite software (version 5.2).

#### Proteomics

Cell pellets were homogenized at a concentration of 1 mg/ml in lysis buffer containing 8 M urea, 50 mM Tris (pH 8.5), protease inhibitor cocktail (Roche Applied Science). Homogenates were allowed to stand at room temperature for 1 h, before being centrifuged at room temperature for 15 min at 13,000 rpm. The supernatant was subjected to reduction, alkylation and quenching with 5 mM DTT, 15 mM iodoacetamide and 15 mM DTT, respectively. Twenty microliter of the resulting solution was diluted five times in trypsin digestion buffer, containing 50 mM Tris (pH 8.5), 5% CH_3_CN, 0.01% ProteaseMAX (Promega) and 0.5 µg modified trypsin (Pierce). After overnight digestion at 37 °C, the samples were brought to 1% TFA and centrifuged for 10 min at 14,000 rpm at room temperature. The supernatant was subjected to desalting with C18 spin columns (Pierce) and 1/15 of the sample was loaded on an Ultimate 3000 UPLC system (Dionex, ThermoScientific) equipped with an Acclaim PepMap100 pre-column (C18, particle size 3 μm, pore size 100 Å, diameter 75 μm, length 20 mm, ThermoScientific) and a C18 PepMap analytical column (particle size 2 μm, pore size 100 Å, diameter 50 μm, length 500 mm, ThermoScientific) using a 4 h linear gradient (300 nL/min) coupled to a Q Exactive Orbitrap mass spectrometer (ThermoScientific) operated in data-dependent acquisition mode. Peptides were identified by Mascot (Matrix Science) using Uniprot Homo sapiens (194619 sequences) as a database. Carbamidomethylation (C) was included as a fixed modification and Oxidation (M) as a variable modification. Two missed cleavages were allowed, peptide tolerance was set at 10 ppm and 20 mmu for MS and MS/MS, respectively. Progenesis software (Nonlinear Dynamics) was used for relative quantification of proteins using the Proteome Discoverer 2.2 Percolator node for peptide validation (FDR < 1%). Proteome data were deposited at ProteomeXchange (PRIDE accession number PXD037976).

### Statistical methods

#### Spearman correlation and differential gene expression

To evaluate gene expression in PDAC from the different studies we used two methods. Spearman rank correlation was used to identify those genes that are statistically strongly linked to pancreatic tumorigenesis and that mechanistically are likely involved in the cancer process. This method uses normalization by standard deviation and Z-score. For each dataset, the results are computed with a range between + 1 (positively correlated = higher expression) and − 1 (negative correlated = reduced expression) with corresponding p-value. In this way, each dataset is normalized to the same scale, which facilitates comparison of different arrays (with variable probes and sensitivity). To identify the strongest molecular markers we also calculated the differential gene expression relative to non-tumor samples within the same dataset. For Spearman correlation and differential gene expression the mean value over the three datasets was computed. For statistical comparison, the median p value was computed for the Spearman correlation, or the t-test value for differential gene expression (expressed as ^10^log value).

#### Hierarchical clustering and pathway analysis

Hierarchical clustering (HC) of the individual samples was done using PermutMatrix program (Version 1.9.3 EN). WebGestalt (WEB-based Gene SeT AnaLysis Toolkit) was used to explore functional enrichment based on KEGG pathways using ranked lists of differentially expressed genes [45]. Statistical analyses (ROC-curve, Spearman correlation) were computed using SigmaPlot 12.3 program. A p-value below 0.05 was considered statistically significant.

## Results

### Establishing a link between cancer, EMT and PP2A in patients

#### EMT gene expression in tumor and non-tumor PDAC samples

**Fig. 1:**
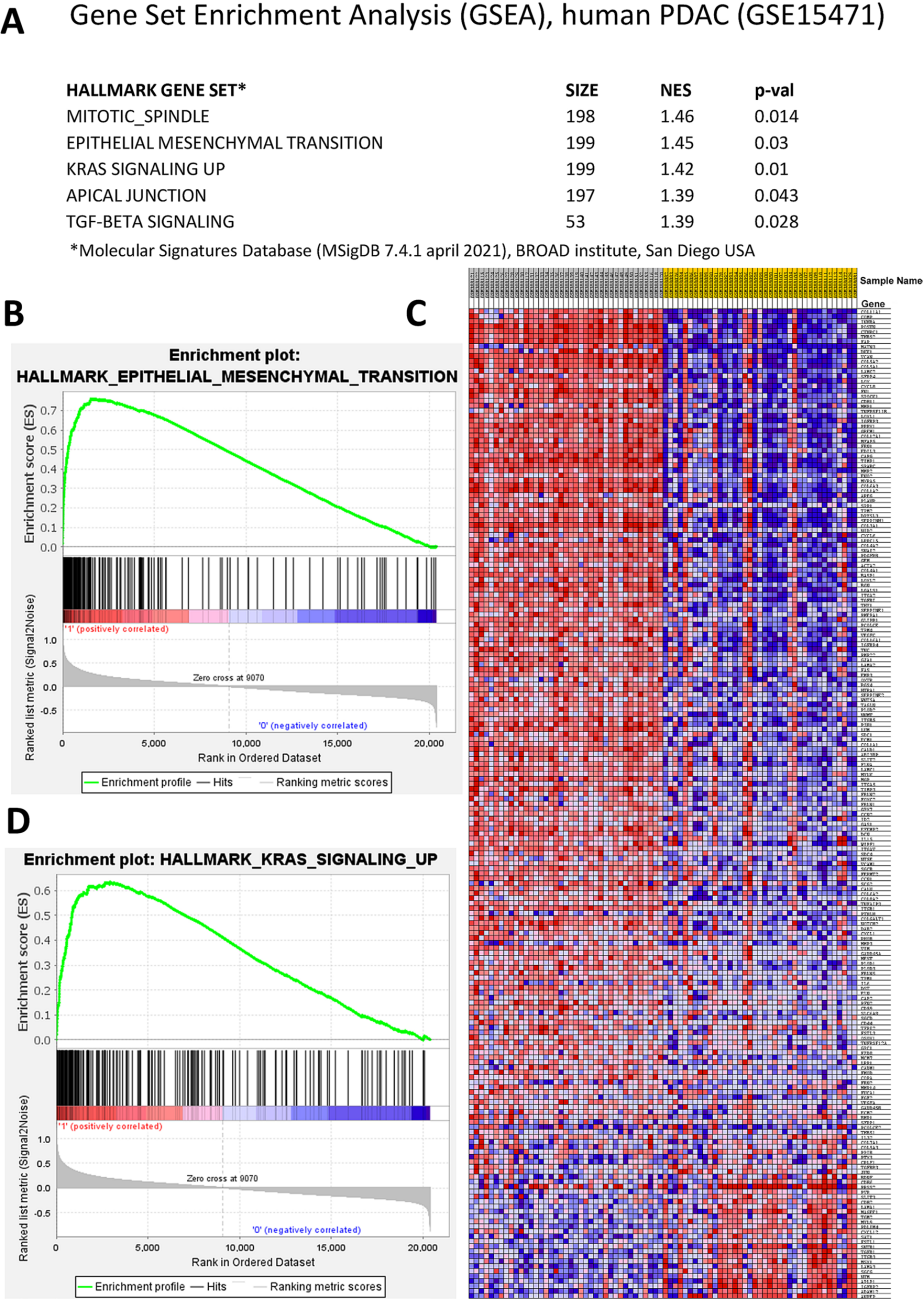
Gene set enrichment analysis of GSE15471 samples. **A** List of top hallmark gene sets enriched in tumor versus non-tumor samples. **B** Enrichment plot for Epithelial-Mesenchymal Transition. **C** Hierarchical clustering of the expression of EMT genes in the hallmark gene set. A color scale was used to visualize the expression: red is for increased and blue indicates reduced expression. **D** Enrichment plot for Hallmark genes upregulated in KRAS signaling. NES = normalized enrichment score. GSEA unsupervised analysis and Enrichments plots were obtained using GSEA software version 4.1.0 (release July 2021) and Molecular Signature Database (MSigDB) 7.4 hallmark gene sets [release April 2021, 13]

To demonstrate the link between PDAC and EMT unsupervised Gene Set Enrichment Analysis (GSEA) for hallmark gene sets (MSigDB 7.4) was performed on the published dataset GSE15471, encompassing 36 tumor and 35 non-tumor samples [5]. In this patient dataset, EMT was found as having the second highest normalized enrichment score (NES) (Fig. 1A), and this was further underscored by the enrichment plot (Fig. 1B) and EMT hierarchical clustering (Fig. 1C). As expected, similar results were obtained for *KRAS* signaling genes, which like EMT genes, are highly enriched in tumor versus non-tumor PDAC (Fig. 1D).

#### Association between EMT and PP2A gene expression: PDAC vs non-tumor tissue

To demonstrate an association in patients between PP2A and EMT, we performed hierarchical clustering in dataset GSE15471 now using a PP2A gene list (Additional file 1: Table S1). We further extended this analysis to two additional at NCBI deposited datasets (GSE16515 and GSE101448) (Table 1). Interestingly, samples were correctly classified as tumor or non-tumor when using the PP2A gene list with a similarly high sensitivity and specificity as was achieved with the IPA (QIAGEN Ingenuity Pathway Analysis)-EMT gene list (Table 1).

**Table 1:** Performance of Hierarchical sample clustering using PP2A or EMT gene set

Study	Samples	PP2A		EMT	
	Tumor/non-tumor (n/n)	Sensitivity (%)	Specificity (%)	Sensitivity (%)	Specificity (%)
GSE15471	36/36	80.6	61.1	86.1	75
GSE16515	36/16	86.1	87.5	80.6	87.5
GSE101448	24/19	79.2	100	100	84.2

Using the clinical information as reported for the individual studies (GSE15471, GSE16515 and GSE101448: tumor = 1 and non-tumor = 0), we next calculated the Spearman correlation for PP2A gene expression vs clinical disease (tumor), and from it, the mean Spearman correlation (SPmean) and median p. This was also done for gene expression relative to non-tumor (meanDelta ^2^log-value) (Table 2). A full list of results for PP2A and EMT-genes in the different datasets is given in the Additional file 1: Table S2.

**Table 2:** Top 10 PP2A-genes and EMT-genes in PDAC-datasets

A: PP2A					
Spearman correlation			Gene expression		
Gene	SPmean	Median p ^10^log	Gene	meanDELTA	t-test Median p ^10^log
*PPP2R1B*	− 0.602	− 7.590	*PPP2R1B*	− 0.637	− 7.082
*SETBP1*	− 0.430	− 5.121	*PPP2R2D*	− 1.056	− 4.393
*KIAA1524*	0.518	− 4.539	*SETBP1*	− 0.652	− 4.327
*PPME1*	0.452	− 3.833	*PPME1*	0.279	− 3.971
*ARPP19*	0.465	− 3.342	*KIAA1524*	0.320	− 3.868
*PPP2R2D*	− 0.547	− 3.048	*ARPP19*	0.416	− 3.443
*PPP2R2A*	0.405	− 2.951	*PPP2R5E*	0.453	− 3.149
*TIPRL*	0.408	− 2.943	*TIPRL*	0.314	− 2.898
*STRN*	0.403	− 2.654	*ANP32A*	0.379	− 2.219
*PPP2R5E*	0.456	− 2.161	*PPP2R2A*	0.275	− 2.163

B: EMT					
Spearman correlation			Gene expression		
Gene	SPmean	Median p ^10^log	Gene	meanDELTA	t-test Median p ^10^log
*KRT19*	0.716	− 10.000	*KRT19*	2.113	− 9.064
*TWIST1*	0.615	− 10.000	*TWIST1*	1.658	− 7.562
*HIF1A*	0.512	− 8.423	*HIF1A*	0.675	− 7.010
*LEF1*	0.565	− 5.963	*LEF1*	1.172	− 6.648
*MMP2*	0.486	− 5.963	*FGFR1*	− 0.658	− 6.158
*IRS1*	0.635	− 5.842	*YAP1*	0.766	− 5.571
*SNAI2*	0.457	− 5.520	*MMP2*	0.828	− 4.946
*YAP1*	0.555	− 5.294	*CTNNB1*	0.405	− 4.582
*FGFR1*	− 0.579	− 5.121	*IRS1*	1.038	− 4.512
*ZEB1*	0.623	− 5.080	*FZD7*	0.949	− 4.414

For dataset GSE15471, GSE16515 and GSE101448, the Spearman correlation was computed, and from these the mean Spearman correlation (SPmean: tumor = 1 vs non-tumor = 0) for the A: PP2A genes and for the B: EMT genes. The median p-value was computed and expressed as ^10^log. Differential gene expression was also computed (tumor vs non-tumor as ^2^log values) and the mean was calculated (meanDELTA). A t-test was computed for the individual genes in each dataset followed by calculation of median p-value (^10^log)

Genes that are upregulated in the tumor have a positive correlation coefficient, whereas genes that are downregulated in PDAC have a negative value (Table 2). We found among the top altered genes a downregulation of *PPP2R1B* and *PPP2R2D* in tumors, and an increase of *PPP2R2A*, *PPP2R5E* and *STRN*. Furthermore, a positive correlation was found for several endogenous PP2A inhibitors (*ARPP19*, *PPME1*, *TIPRL* and *KIAA1524/CIP2A*). EMT genes like *KRT19*, *CTNNB1* and *TWIST1* and the hypoxia marker *HIF1A* were significantly changed in tumors, indicating altered tumor microenvironment. The changes of EMT gene expression in tumor were in general more pronounced than for PP2A. The Spearman correlation and differential gene expression showed similar results. Differences found between the gene sets can further be ascribed to the selection of patients and the expression platform used for analysis.

**Fig. 2:**
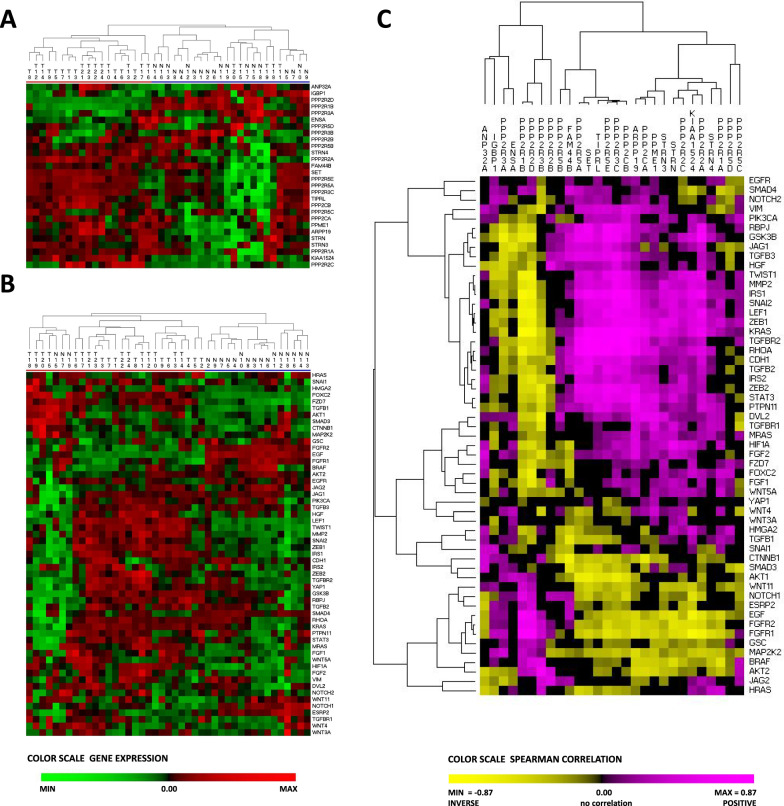
Correlation between PP2A genes and EMT genes in tumor vs. non-tumor pancreatic cancer (GSE101448). **A** Hierarchical clustering (HC) of gene expression for the PP2A genes was performed on tumor samples (T_xx) and non-tumor samples (N_xx). The samples were separated into two clusters indicated at the top; red (enriched for tumor) and blue (enriched for non-tumor) with a sensitivity of 79.2% and a specificity of 100%. The color scale illustrates the relative level of gene expression (Z-score): red, increased expression above the mean Z-score; green, reduced expression in tumor. **B** HC was performed using the EMT gene expression, which also resulted in two clusters; red (enriched for tumor) and blue (enriched for non-tumor) with a sensitivity of 100% and a specificity of 84.2%. **C** The Spearman correlation was computed for each gene in the PP2A gene list to each of the EMT genes. These correlations are visualized in an HC-graph showing distinct clustering. To make a distinction with gene expression, the Spearman correlation is indicated with a color scale ranging from yellow to pink; clusters indicate genes that are co-regulated in PDAC

Hierarchical clustering of GSE101448 for PP2A or EMT genes is shown in Figs. 2A, B, again demonstrating significant clustering of specific PP2A or EMT genes with tumor vs. non-tumor tissue. To explore a direct link between PP2A and EMT expression, we also calculated the Spearman correlation between individual PP2A genes and EMT genes. We present these results by hierarchical clustering: the graph clearly shows clusters, indicating important co-regulation of the signaling for PP2A and EMT in patients (Fig. 2C).

#### Expression of PP2A genes as markers in advanced disease

**Fig. 3:**
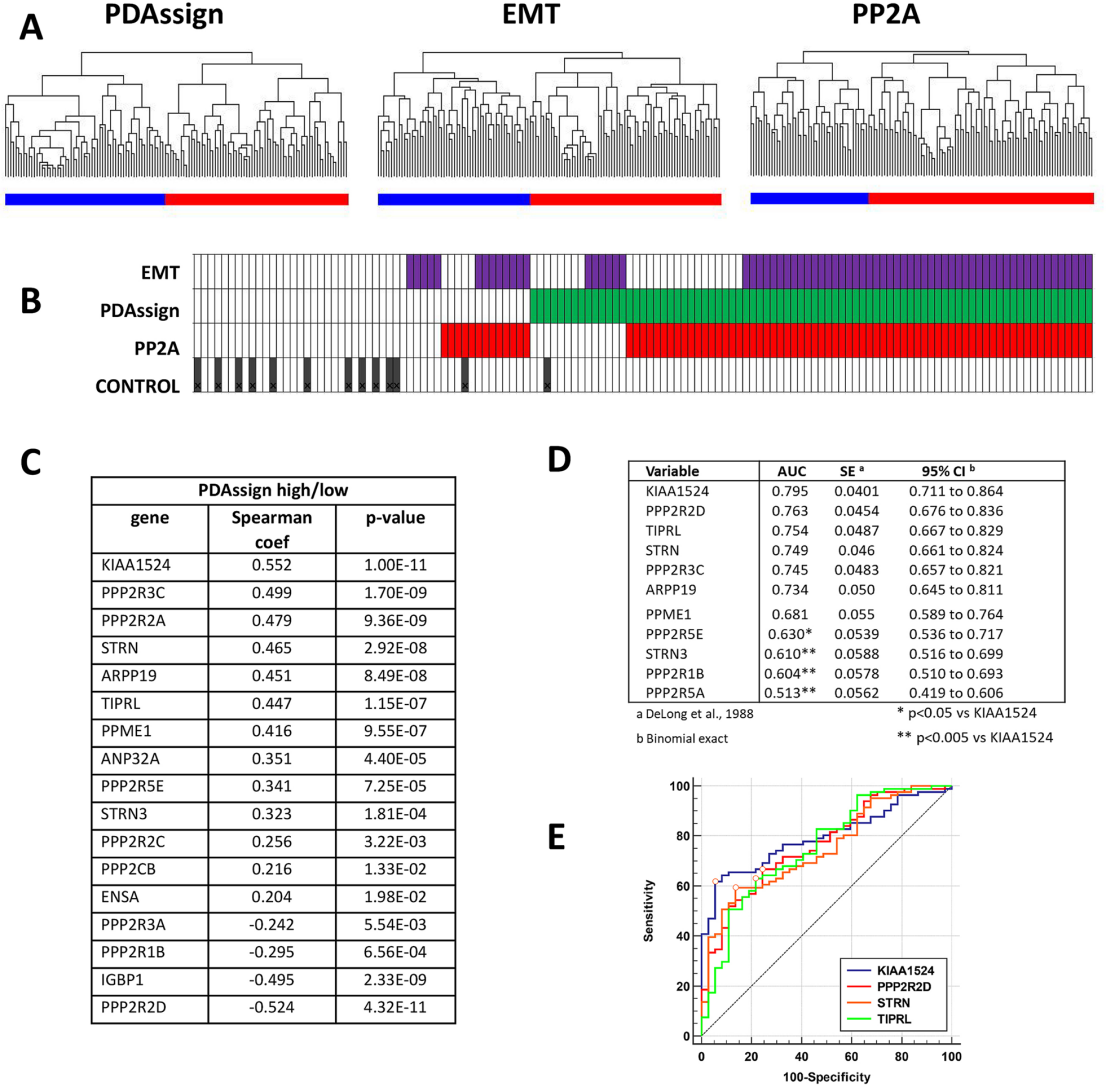
Molecular classification of PDAC patients using PP2A-related genes in advanced disease (GSE62165 dataset). **A** Tumor samples were clustered based on PDAssign, EMT or PP2A gene sets (see Additional file 1: Table S1), each classifier resulted in two clusters. **B** Parallel alignment of samples using the different classifier. Advanced disease (PDAssign-high) samples are indicated in green. Using the corresponding EMT-high classification these same samples are marked in purple. The PP2A-high clustered samples are indicated in red. The PDAssign-low/EMT-low or PP2A-low samples are indicated in white. The GSE62165 dataset included 13 control normal pancreatic samples indicated with the black bars. **C** Spearman correlation of PP2A-related genes with molecular identified subgroup of PDAC patients with worse prognosis (PDAssign-high [43, 46]). **D** Receiver operating characteristic (ROC) analysis of the patient cohort. Patients were clustered into two groups using PDAssign 62 gene set. AUC (Area under the ROC Curve) for individual PP2A-related genes that cluster into PDAssign-high group was calculated based on gene expression with SE (standard error) and CI (confidence interval). **E** Graphic presentation of ROC-analysis for selected markers

In the period 1998–2010, we collected tissue samples from 118 surgically resected PDAC and 13 histologically normal pancreatic tissue samples and performed gene expression analysis (dataset GSE62165) [43]. Using the PDAssign gene set in this study, hierarchical cluster analysis had identified two clusters with statistically significant different survival [43] (Fig. 3A). The EMT gene set also achieved a separation into two groups (Fig. 3A) with the worse prognosis for disease-free-survival and overall-survival for EMT-high [46].

Interestingly, when applying this methodology to the PP2A genes, there was also a separation into two groups: the PP2A-high group strongly overlapped with the PDAssign-high and the EMT-high group (Fig. 3B). Using the two PDAssign clusters ‘high/aggressive’ vs. ‘low/moderate’ PDAC, we then further investigated the association of individual PP2A genes with worsened prognosis in PDAC. We found a significant association for various PP2A regulatory subunit encoding genes, including *PPP2R3C, PPP2R2A, STRN* (all upregulated) and *PPP2R2D* and *PPP2R1B* (both downregulated), as well as for PP2A-inhibitors *KIAA1524/CIP2A, ARPP19*, and *TIPRL* (all upregulated) with more aggressive PDAC (Fig. 3C, Spearman p < 0.05). Thus, the expression balance between the different PP2A genes is significantly shifted in aggressive PDAC.

To further assess clinical importance, we investigated whether PP2A genes could be markers for the prediction of an aggressive PDAC phenotype. Using the PDAssign clusters as a measure for malignancy, Receiver Operating Characteristic (ROC) analysis returned that the highest performance was obtained with the PP2A inhibitor *KIAA1524/CIP2A* (Fig. 3D, E). Other PP2A-inhibitors (*TIPRL, ARPP19* and *PPME1*) were also predictive for a more aggressive PDAC, as were some PP2A regulatory subunits such as *PPP2R2D*, *PPP2R3C* and *STRN* (Fig. 3D, E).

#### Clinical evaluation of PP2A proteins in tissue samples (Clinical Proteomic Tumor Analysis Consortium: CPTAC) and of gene expression in TCGA samples

**Fig. 4:**
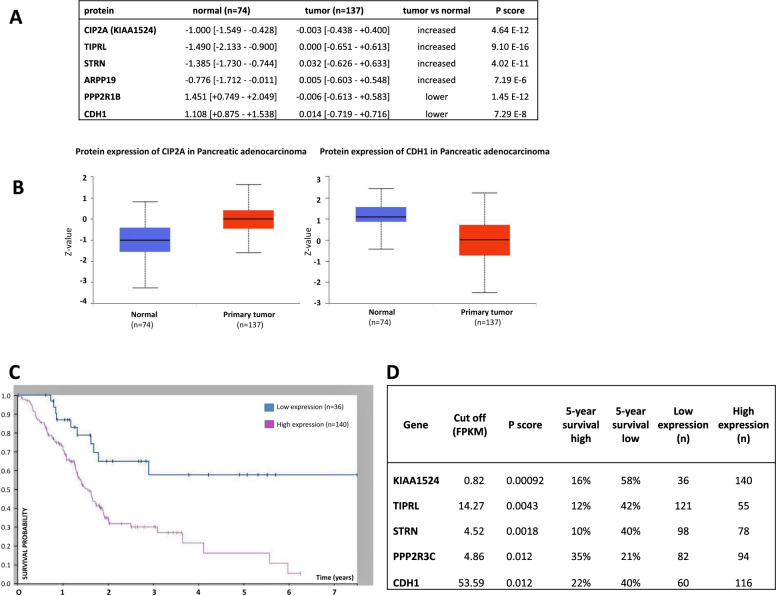
Clinical evaluation of PP2A-markers in Clinical Proteomic Tumor Analysis Consortium (CPTAC) samples and TCGA samples. **A** Protein expression of top PP2A genes identified in the GSE62165 patient samples. Log2 Spectral count ratio values from CPTAC were first normalized within each sample profile and then normalized across samples. Z-values represent standard deviations from the median across samples for the given cancer type. **B** Graphs showing protein expression for CIP2A and CDH1. **C** Gene expression in TCGA dataset, Log-rank graph for overall survival for *KIAA1524/CIP2A* of PDAC patients. **D** Survival analysis in patients. Log-rank overall survival analysis of TCGA dataset of PDAC patients for selected PP2A genes and *CDH1* (representing EMT). (FPKM = Fragments Per Kilobase of transcript per Million mapped reads). (TCGA: access August 13, 2022 via https://www.proteinatlas.org/ENSG00000163507-CIP2A/pathology/pancreatic+cancer)

Since PP2A and its inhibitors exhibit their interplay at the protein level, we further assessed protein expression in patients of tumor vs. non-tumor tissue in the CPTAC dataset [41]. Proteins encoded by PP2A genes identified to be highly correlating with aggressive PDAC in GSE62165 were also significantly different between normal tissue and PDAC tumor, with again CIP2A, TIPRL and STRN as top altered hits (Fig. 4A, B). Reassuringly, E-cadherin (encoded by the EMT-gene *CDH1*) was found significantly down-regulated in tumor vs. normal tissue, a prototype marker for EMT.

In the TCGA dataset, analyzing overall survival for RNA, the top results from CPTAC were confirmed with high statistical significance for *KIAA1524/CIP2A, TIPRL* and *STRN* (Fig. 4C, D)—further strengthening our findings from the four datasets (GSE15471, GSE16515, GSE62165 and GSE101448) presented before.

### PP2A inhibition shifts EMT under experimental conditions

#### Gene and protein expression in human Panc-1 cells upon short exposure to Okadaic acid

In patients, we found a close correlation between aggressive PDAC on the one hand, and both EMT-induction and dysregulation of PP2A on the other hand. However, these results did not tell us which factor could be the driver, and which factor could be the consequence.

**Fig. 5:**
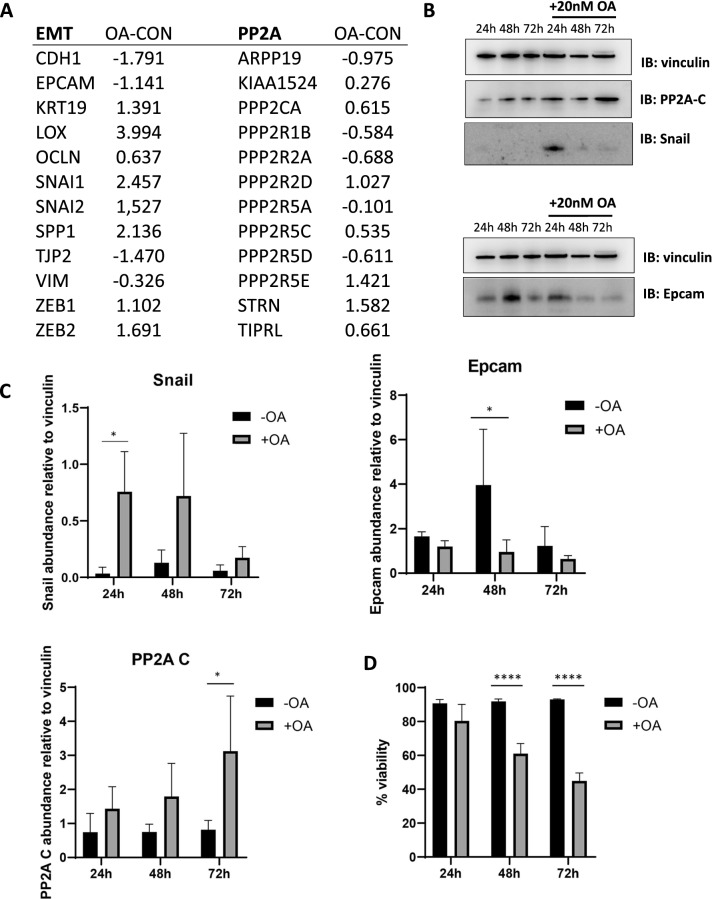
Short-term inhibition of PP2A by Okadaic acid drives changes in gene and protein expression in Panc-1 cells. **A** Relative gene expression of selected EMT and PP2A genes in Panc-1 cells treated with OA for 24 h vs untreated control cells (GSE114288) (^2^log values). **B** Western blot analysis of Panc-1 cells exposed to 20 nM of OA for 24, 48 or 72 h. Expression is shown for vinculin (loading control), PP2A-C, Snail and Epcam. A representative blot is shown. **C** Protein bands were quantified and relative changes in protein expression were determined relative to vinculin for three independent immunoblot experiments. Average values for n = 3 are displayed; p-values < 0.05 were considered significant (*, two-way ANOVA). **D** Average percentage viability of Panc-1 cells treated with ethanol (mock) or 20 nM OA for 24 h, 48 h or 72 h as measured with trypan-blue on an automated cell counter (n = 3). p-values < 0.05 were considered significant (two-way ANOVA, ****p < 0.0001)

To elucidate this, we performed data analysis and in vitro studies in which we used Okadaic acid (OA: a known selective pharmacologic inhibitor of PP2A) in the human pancreatic cancer cell line Panc-1. First, we analyzed the expression data of Panc-1 treated for 24 h with OA by RNAseq in a published study (GSE114288) [47]. We found that short-term PP2A inhibition with OA induced markers of EMT (e.g., Snail) and reduced epithelial markers (e.g., E-cadherin and Epcam), and modulated expression of some PP2A subunits or inhibitors at the gene expression level (Fig. 5A). Complementarily, we performed Western blot analysis of Panc-1 cells incubated for 24 h, 48 h or 72 h with 20 nM OA, which mirrored the shifts for EMT and PP2A proteins that were seen at the RNA level, with the transcription factor Snail responding strongly at 24 h and the others (PP2A-C and Epcam) with some delay (Fig. 5B, C)—while no significant changes in cell viability were observed at 24 h under these conditions (Fig. 5D). In contrast, incubation of Panc-1 cells with TGF-β1 (48 h, 5 ng/ml, which induced EMT) [48] had limited effect on PP2A gene expression (GSE23952, data not shown [49]).

Overall, these data thus suggest that a general inhibition of PP2A (as seen by OA in Panc-1) or a disturbed PP2A balance (as seen in the patient samples) may actually drive EMT, and thereby, aggressive PDAC.

#### Long-term exposure model of PP2A-inhibition in human pancreatic cancer cells

**Fig. 6:**
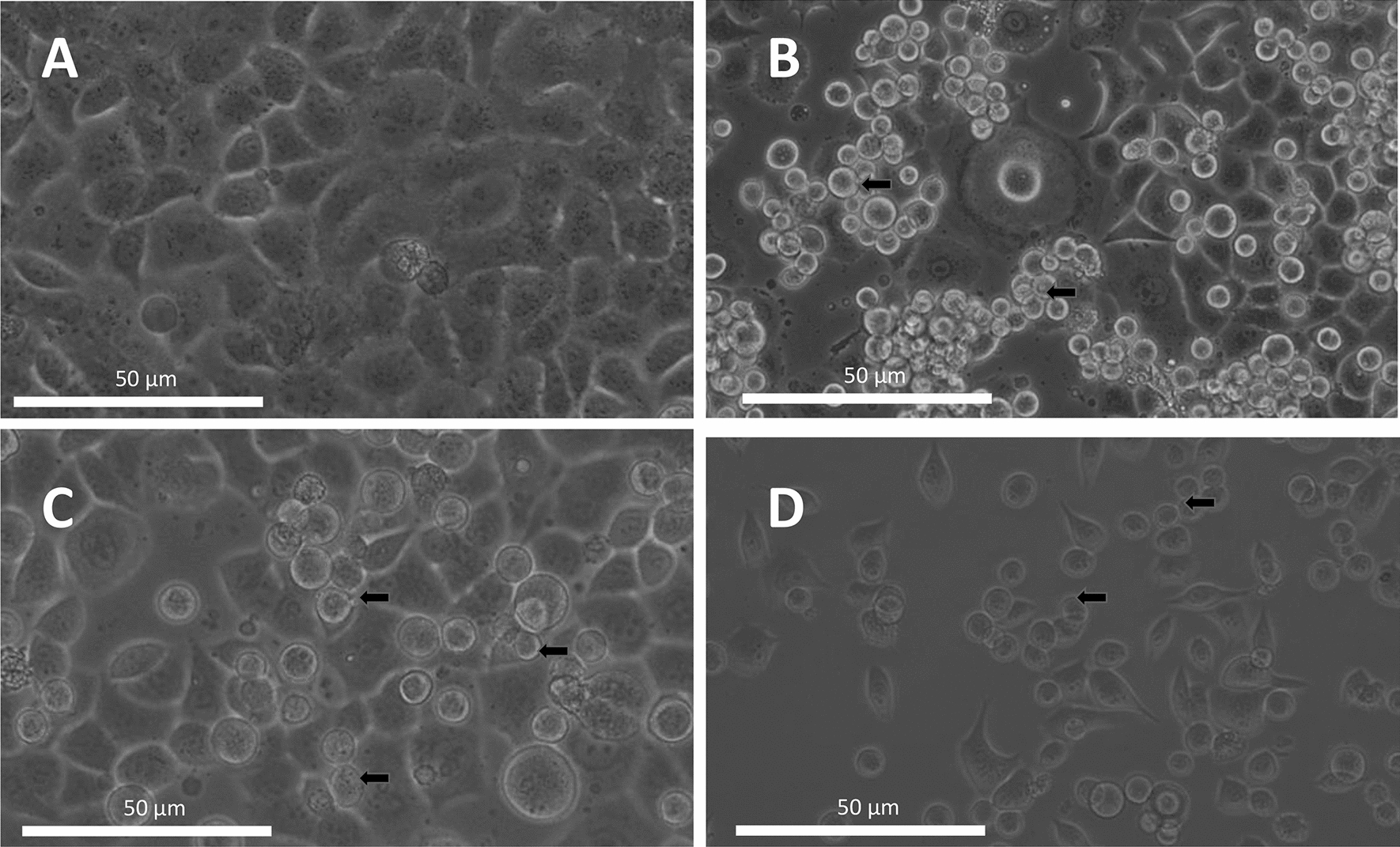
Morphological appearance of control Panc-1 and different Okadaic acid resistant clonal cell lines. **A** Panc-1 control cells, **B** OA7A cells, **C** OA7G cells and **D** OA7H cells. Cell lines were developed to monoclonal over a period of 3–6 months as described in the materials & methods section. The resistant cells showed loss of cell-to-cell contact (arrows) (original magnification ×200)

As these in vitro results seemed to suggest a causal relationship between PP2A inhibition and induction of EMT, we next developed a PDAC-model based on continuous exposure of Panc-1 cells to OA to better mimic the cellular response to structural, long-term dysregulation of PP2A, which might occur during tumor progression. Four OA-resistant cell lines were developed to single clones following serial dilution: OA7A, OA7G, OA7H and OA10G (each clone can grow and multiply in the presence of 8 nM OA). Morphologically, resistance to long-term exposure to OA resulted in partial loss of dependence on cell–cell contact, as many cells have a round appearance but are not dead (arrows) and others show a more elongated, appearance suggestive for cells that have undergone EMT (Fig. 6A–D).

#### Molecular characterization of Okadaic-resistant (PP2A-inhibited) pancreatic cells

From these, we selected the OA7G and OA7A cell lines for further characterization by RNA seq (GSE216036, this study). These data were used for comparison with the RNAseq profile of short-term treated Panc-1 cells (24 h: GSE114288) and to perform pathway analysis, in order to get insight in the processes induced by acute versus chronic PP2A inhibition in Panc-1 cells (Table 3).

**Table 3:** Top enriched pathway identified by WebGestalt 2019 [45]

Panc OA24hr	geneSet	Description	NES	FDR
	hsa01200	Carbon metabolism	− 2.26	0.000
	hsa05134	Legionellosis	2.10	0.000
	hsa04657	IL-17 signaling pathway	2.14	0.001
	hsa00020	Citrate cycle (TCA cycle)	− 2.04	0.002
	hsa00620	Pyruvate metabolism	− 2.04	0.003
	hsa01212	Fatty acid metabolism	− 2.05	0.004
	hsa00670	One carbon pool by folate	− 1.95	0.004
	hsa00640	Propanoate metabolism	− 1.96	0.005
	hsa05323	Rheumatoid arthritis	1.92	0.005
	hsa04668	TNF signaling pathway	1.93	0.005

**OA7G**	geneSet	Description	NES	FDR
	hsa04062	Chemokine signaling pathway	1.71	0.123
	hsa00730	Thiamine metabolism	− 1.73	0.137
	hsa05231	Choline metabolism in cancer	1.71	0.153
	hsa04512	ECM-receptor interaction	1.74	0.155
	hsa04510	Focal adhesion	1.76	0.219
	hsa05214	Glioma	1.64	0.235

The immediate effect of OA (24 h: GSE114288) appeared a strong downregulation of metabolism and an upregulation of pathways involved in acute and chronic inflammatory response (Table 3). However, when Panc-1 cells were cultured continuously in the presence of OA, other pathways appeared that are related to EMT (Focal adhesion and ECM-receptor interactions) (Table 3). In addition, twenty-four-hour culture with OA had a stronger effect on gene expression (6300 of 12,400 genes showed altered expression with a ^2^log >  + 1 or < − 1) than long-term culture (only 2749 and 2863 genes for clones 7A and 7G respectively showed altered expression—see also Fig. 5A vs Additional file 1: Table S3), suggesting adaptation over time.

**Fig. 7:**
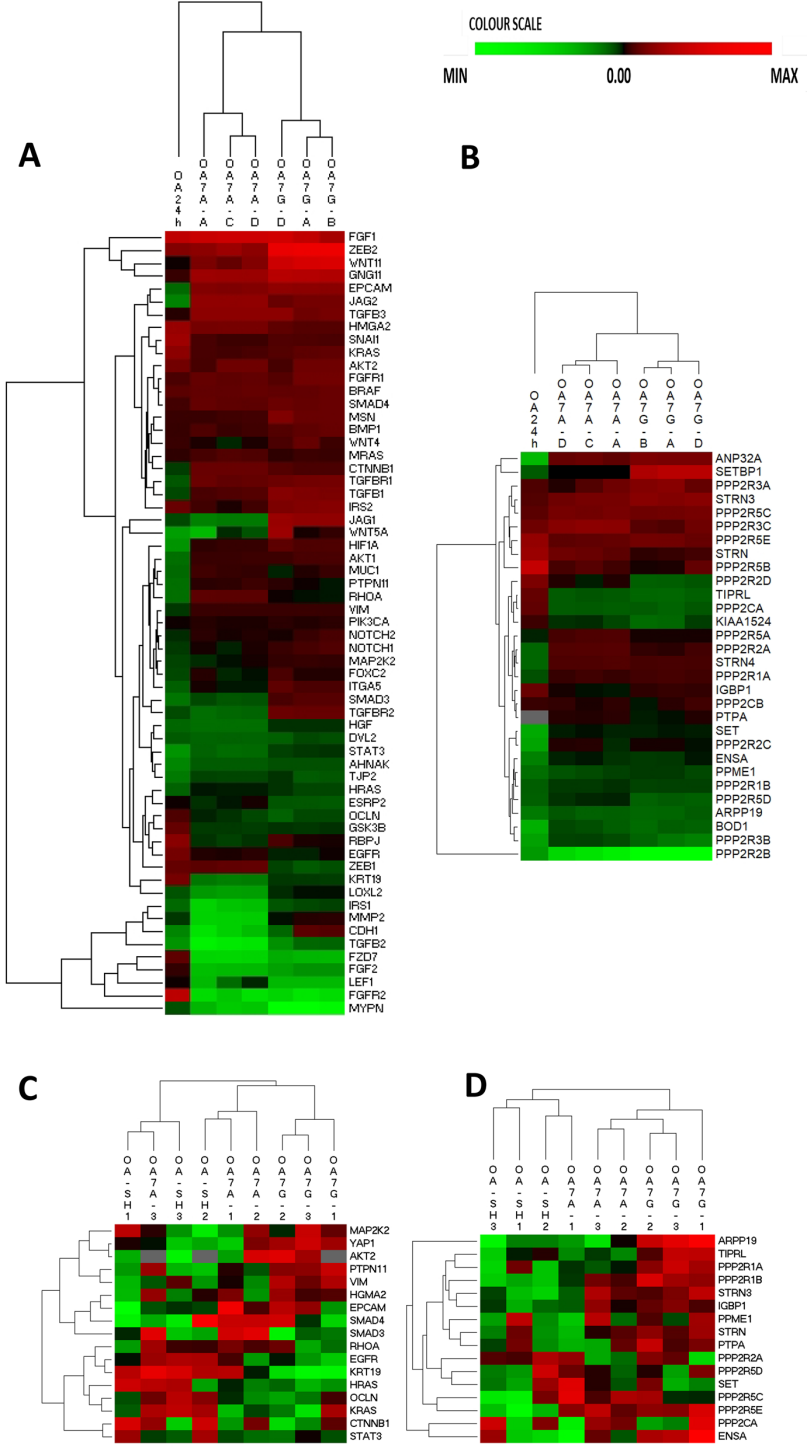
Gene expression and proteomics analysis of Panc-1 cells exposed to Okadaic Acid. **A** Cells were exposed to 20 nM of Okadaic Acid for 24 h (OA-SH) and OA resistant clones OA7A and OA7G were analyzed. For EMT gene expression we compared to the published data for OA24h (treated vs untreated: GSE114288). Gene expression of clones was relative to three independent untreated samples. **B** PP2A-gene expression. **C** EMT protein expression; control and OA-treated Panc-1 cells were analyzed in triplicate. Changes were determined relative to untreated Panc-1 cells as described in the materials and methods section. **D** PP2A proteins that were detected in the output list. Hierarchical clustering was used to present the levels. A color scale was used to visualize the expression: red is for increased and green indicates reduced expression versus the untreated control, grey: not present

Specifically, significant expression changes of several EMT-related genes (e.g., *AKT2, FGF1, JAG1, WNT11, TGFB1, ZEB2*) were observed in both short- and long-term OA-treated conditions, compared to the naïve Panc-1 cells (Fig. 7A). The EMT gene expression signatures of OA7G and OA7A were, although not identical, still largely overlapping, while they significantly differed from the EMT gene signature found in Panc-1 cells exposed to OA for only 24 h. Notably, e.g., expression of *EpCAM, CTNNB1* and *KRT19* changed in opposite directions in short-term vs. long-term treated cells, whereas e.g. *ZEB2, SNAI1* and *AKT2*, responded in the same way (Additional file 1: Table S3, Fig. 7A).

Intriguingly, OA also affected expression of several PP2A genes (Fig. 7B). Compared with untreated cells, especially *PPP2R2B* was strongly suppressed, while other members such as *PPP2R3C* and *STRN* appeared strongly upregulated in OA-treated cells. In addition, as observed for the EMT genes, some PP2A genes were oppositely affected in short- versus long-term treated conditions (e.g., *PPP2CA, PPP2R1A* and many of the endogenous PP2A inhibitor genes) (Fig. 7B) (detailed numbers shown for OA7G in Additional file 1: Table S3).

To further complement these observations with what happens at the protein level, we performed proteomics analysis on Panc-1 cells treated with OA for 24 h (OA-SH, n = 3) and in both OA-resistant clones (OA7A, n = 3 and OA7G, n = 3). We anticipated that the protein expression pattern might differ from the gene expression and could provide insight into the complexity of balanced regulation of PP2A enzyme activity [50]. We were able to identify 3780 proteins throughout all datasets (see Materials and Methods and protein dataset PXD037976). Of these, the proteins present in the EMT list and those from the PP2A list were selected and subsequently analyzed by hierarchical clustering (Fig. 7C, D—statistical overview in Additional file 1: Table S4).

At the protein level, we found limited effects on PP2A expression in the cells treated for 24 h (OA-SH), while a shift becomes more pronounced in clone OA7G, and clone OA7A showed an intermediate change in PP2A protein expression pattern (Fig. 7D and Additional file 1: Table S4A). For the EMT markers, each of the conditions has its own protein expression pattern (Fig. 7C and Additional file 1: Table S4B). Differential expression evaluation versus untreated cells confirmed the clustering, while, between the clones, no statistical difference was identified (Additional file 1: Table S4). Protein expression (mass spectroscopy) and gene sequencing showed that the advanced model OA7G most resembles the clinical PDAC presentation (with EMT markers, *PPP2R3D, PPP2R5E, ARPP19* and *TIPRL* upregulated, and *PPP2R2D* downregulated). Under permanent exposure to OA, the role of endogenous PP2A inhibitors is not as pronounced in these models.

## Discussion

Epithelial-to-Mesenchymal Transition is frequently occurring in different types of tumors. EMT is a concept that has recently become better understood and appears to have major clinical impact in tumor progression as it can be linked to several aspects of aggressive cancer, drug resistance and invasion [12]. We could indeed confirm EMT in aggressive PDAC in multiple patient cohorts (Figs. 1, 3A, B and Table 1). However, the relationship between PP2A and EMT in PDAC patients remained to be explored.

PP2A phosphatases are generally considered as tumor suppressors, although some PP2A complexes, characterized by defined B-type subunits, have been attributed oncogenic functions and again others can play either role depending on the tumor context [20, 51]. It remained a question how their role, linked to subunit composition of their complexes, shifts from normal cells to cancer in PDAC and in corresponding EMT. In cell line models and xenografts of gastric cancer [52], lung cancer [53] and prostate cancer [54] modulation of EMT and PP2A signaling has been studied. In vitro, it was shown that the RHOB/PP2A/AKT1/RAC1 pathway regulates mesenchymal migration and invasion in lung cancer through implication of a PP2A-B55 complex [55]. Furthermore, the B55α subunit of PP2A showed higher expression in PDAC compared to adjacent normal tissue, and in mouse models, it supported the tumorigenic and metastatic potential of pancreatic cancer cells [27], in line with our own observations. Overexpression of CIP2A in PDAC [30], clear cell renal cell carcinoma [56] or bladder cancer [57] was shown to promote cellular EMT and was associated with poor prognosis. Also, both SET and SETBP1 have been implicated in EMT [31, 32].

In the PDAC patient cohorts we studied, and that were collected at different institutes and with gene expression determined by different methods, several associations for PP2A were found. Three of the studies investigated reported on tumor tissue vs non-tumor tissue (GSE15471, GSE16515 and GSE101448), and in all these, we found an upregulation of *KIAA1524/CIP2A, TIPRL, STRN* and *ARPP19*, and a reduced expression of *PPP2R1B* in the tumor. Under conditions when we had a confirmed PDAC and we classified the tumors according to the Collisson gene set, we also found a significant correlation for a number of PP2A genes with PDAC of a mesenchymal phenotype. EMT-high PDAC showed suppression of *PPP2R1B* and *PPP2R2D* gene expression and upregulation of *PPP2R3C, STRN, STRN3* and *PPP2R2A*. The strongest link was found for upregulation of endogenous inhibitors of PP2A enzyme activity (*KIAA1524/CIP2A, ARPP19, TIPRL, PPME1* and *ANP32A*), each of which may affect specific subsets of PP2A holoenzymes [22, 23, 58]. Finally, in our study, the ROC-curve indicated that high expression of PP2A-inhibitors *CIP2A, TIPRL, ARPP19* and *PPME1* are predictors of aggressive PDAC. Also, EMT is a predictor of aggressive PDAC. Our study thus found evidence for a number of PP2A genes to be linked to either tumor induction or tumor progression. Especially the strong correlation of increased expression of PP2A inhibitors with a more aggressive tumor makes regulation of PP2A enzymatic activity a clinically relevant target for cancer treatment.

To be noted, for technical reasons, not all genes were detected above the background in each study, resulting in variable numbers of genes reported. A second factor that could influence the statistical results in our study is the selection criteria used for the patient cohorts (tumor criteria and treatment history). We used Spearman correlation to identify genes that are most strongly linked to PDAC and thereby related to the mechanism. Differential gene expression is better suited to identify potential markers for PDAC. Due to the high number of genes involved in the combinatorial assembly of PP2A holoenzymes, our analysis might also not identify all ‘PP2A’ factors involved in PDAC. What our results nevertheless clearly demonstrate is that changes in PP2A expression are highly associated with EMT-high (aggressive) PDAC.

In order to develop a model in which we could study the PP2A-EMT link, we incubated Panc-1 cells for a short time (24 h) to the PP2A-inhibitor OA and made Panc-1 clones resistant to OA. Analysis indicated that short-term induction by OA induced a strong effect on expression of genes involved in metabolism and immunological pathways, while a limited shift was observed on overall EMT and PP2A protein and gene expression (Table 3). When we evaluated expression of select EMT genes in acutely OA-treated cells, we did nevertheless observe specific expression alterations that were consistent with induction of EMT (Fig. 5), while EMT induction in Panc-1 cells with TGF-β1 did not trigger prominent changes in PP2A gene expression (see dataset GSE23952). In the OA-resistant clones, we observed a profound shift of pathways involved in focal adhesion, EMT and ECM-receptor interaction (Table 3), corresponding to the morphological changes seen in the cells (Fig. 6). As, PP2A, has not been assigned a specific KEGG pathway, it did not come forward in this pathway analysis. In the resistant cell lines, the pattern for RNA that was seen after 24-h-exposure stabilized, with some markers becoming more outspoken while others lost their significance versus the control cells (Fig. 7A and B), suggesting some form of adaptation. The gene expression pattern of OA7A and OA7G was largely the same (Fig. 7A and B). At the protein level however there appeared more differences between the clones, suggesting that OA7G is in a more EMT and PP2A advanced cancer stage (Fig. 7C, D). Also, in this model changed expression of the endogenous inhibitors (*ARPP19, TIPRL, PPME1*) and cellular activators (*IGBP1, PTPA*) was found associated with the EMT induction in OA7G, although it remains to be further investigated whether this contributed to EMT induction, or rather presented an adaptation of the PP2A system to chronic inhibition by OA.

Our OA7G model gave a first indication that dysregulation of PP2A precedes EMT and that therefore a sequence of “TGF-β =  > induced EMT =  > induced PP2A” is unlikely. We can only speculate about the molecular mechanisms by which the PP2A genes may affect EMT in pancreatic cells. *PPP2R2A* (B55α) and *PPP2R2D* (B55δ) have been shown to play opposite roles in TGF-β/Activin/Nodal signaling, a major EMT-inducing pathway: knockdown of B55α inhibited this pathway, whereas knockdown of B55δ enhanced the TGF-β-induced responses [59], in line with our observations of increased *PPP2R2A* and decreased *PPP2R2D* expression in EMT-high PDAC. The increased expression of the striatin subunits *STRN* and *STRN3* in EMT-high PDAC may be explained by their role in Hippo signaling, where they counteract MST1/2 kinase activation and YAP1 downregulation as part of the STRIPAK complex [51, 60]. In PDAC, activated YAP1 has been associated with EMT plasticity and metastasis induction [28]. In addition, B55α (*PPP2R2A*) may also contribute to YAP1 activation in PDAC by suppressing Hippo signaling [29]. Although a positive role for the PP2A B”α/PR130 subunit (encoded by *PPP2R3A*) has been inferred in cancer cell migration [61], the role of the related B”γ/G5PR subunit (encoded by *PPP2R3C*) in migration or EMT induction remains unclear. The increased expression of the PP2A B56ε subunit (encoded by *PPP2R5E*) observed in EMT-high PDAC, might be linked to its functional role in the dephosphorylation of a negative regulatory site on KSR-1 through which it promotes MAPK signaling downstream of RAS, and for which it was shown that this pathway contributes to increased growth of PDAC xenografts in mice [62]. As already explained above, *PPP2R1B* also specifically contributes to regulation of a signaling pathway downstream of RAS, by decreasing RalA phosphorylation and activity [63]: thus, decreased *PPP2R1B* expression may contribute to increased cancer cell migration and EMT-high state. Likewise, many of the cellular PP2A inhibitors may contribute to enhance RAS signaling. For example, bioinformatics analysis of RAS and CIP2A-regulated phosphoproteomes revealed a significant overlap in their functional pathways, and strong synergies between CIP2A and RAS expression or *KRAS* mutation in cancer cell growth and survival were found [64]. Interestingly, PME-1- and SET-regulated phosphoproteomes in part overlapped, in part differed from CIP2A-regulated proteomes, indicating they likely target different PP2A complexes, and their functions do not entirely overlap [65]. Clearly, however, PME-1 overexpression has been strongly linked with increased ERK signaling, another pathway downstream of RAS [65, 66]. Since nearly all PDACs have activating *KRAS* mutations, the latter observations may be of high relevance to rationalize the importance of PP2A dysfunctions in PDAC initiation and progression that we exposed in the current study.

It is now increasingly realized that effective treatment of cancer should involve more than a single therapeutic target. Combinations that include targeted therapy aimed at the tumor cells, the immune cells and at the stroma/micro-environment are at present the major areas. EMT has been investigated as target for cancer treatment and several drugs are now under clinical evaluation [review 67]. Also PP2A stimulation has been suggested as an additional therapeutic target for the treatment of PDAC [38, 68]. In recent years, several molecules have been developed shown to stimulate PP2A by different mechanisms, including the promotion of the selective engagement of specific PP2A holoenzymes [69] and the inhibition of specific cellular PP2A inhibitors [18, 23, 26, 39]. Besides in PDAC [38, 68] some progress has already been achieved with these molecules in several treatment-resistant forms of cancer, including *KRAS*-mutant and TKI-resistant non-small cell lung cancer [37, 70], castration-resistant prostate cancer [71], endometrial cancer [72], leukemia [38, 73–75] and neuroblastoma [76]. In a recent review, the involvement of PP2A with aberrant epigenetic states, EMT and cellular plasticity was explored, and this study also suggests to target PP2A to suppress tumor growth [77]. To be noted: PDAC are especially difficult to treat due to their microenvironment [10–12]. Nanoparticles may show potential to overcome some of these therapeutic barriers and this should be considered when designing new therapies [78].

## Conclusions

We could demonstrate a strong association of EMT with altered PP2A expression in pancreatic cancer patients. Although in absolute values the PP2A-genes are not very strongly altered in PDAC, their high statistical correlation strongly suggests their clinical importance. Pharmacologic inhibition of PP2A by Okadaic acid induced EMT in human pancreatic cells in vitro. Two cell models with chronically inhibited PP2A were developed with different stages of EMT. When we examined these models more closely, we found statistically significant changes in protein expression of cellular PP2A inhibitors in the more advanced OA-induced EMT model (OA7G). In EMT-high patient samples, gene expression of natural PP2A inhibitors is also significantly upregulated, suggesting their causal involvement in driving EMT in aggressive PDAC tumors. Unfortunately, this aspect of PP2A inhibition by its naturally occurring cellular inhibitors is not fully captured in our pharmacological model as it works via the same arm of the mechanism. The pathways behind PP2A-inhibition-induced EMT need to be further examined. Translationally, our observations indicate that PP2A is a central factor in PDAC initiation and progression. Therefore, with the development of the new compounds that can target (restore) PP2A enzyme activity, it is attractive to evaluate these compounds in the setting of (combination) therapy for PDAC.

## Supplementary Information

**Additional file 1:** **Figure S1A.** Non-comprehensive overview of major growth signaling functions of PP2A complexes. A key mechanism by which PP2A exerts its tumor suppressive role resides in controlling several components downstream of RAS signaling, including PI3K/AKT, MEK/ERK and JAK/STAT, thereby preventing anomalous cell survival, growth and migration. PP2A is also more directly involved in preventing activation of diverse proto-oncogenes, including β-catenin and c-MYC, as well as in promoting activation of major tumor suppressors, such as p53 and pRb. Other established roles of PP2A occur in regulation of apoptosis, autophagy, cell division and protein synthesis. **Figure S1B.** Structural diversity of PP2A complexes, and their regulation by cellular inhibitors and activators. Functional PP2A complexes consist of one C and one A subunit (each encoded by two different human genes), or one C, one A and one B-type subunit (encoded by 15 different human genes, some of which give rise to multiple isoforms). Additional regulation of phosphatase activity or holoenzyme assembly occurs by endogenous cellular PP2A inhibitory proteins, such as SET, ANP32A, TIPRL, CIP2A, PME-1/PPME-1 and ARPP19, and cellular PP2A activators, such as IGBP1 and PTPA/PPP2R4. **Table S1.** Genes and search terms that were used to investigate the expression datasets: PP2A-search terms, PDAssign and IPA-EMT. **Table S2.** Spearman correlation and differentially gene expression for GSE15471, GSE16515, GSE101448 and calculated means. Legend: **Table S2A.** For dataset GSE15471, GSE16515 and GSE101448, the Spearman correlation (SP) and corresponding p was computed for the PP2A-genes. The mean Spearman correlation (SPmean: tumor = 1 vs non-tumor = 0) and the median for p-value was computed and expressed as ^10^log. **Table S2B.** Differential gene expression (DELTA) was also computed (tumor vs non-tumor as ^2^log values) and the mean was calculated (meanDELTA). A t-test was computed for the individual gens in each dataset followed by calculation of median p-value (^10^log). **Table S2C.** Spearman correlation for EMT-genes. **Table S2D.** Differential gene expression for EMT genes. The in this paper used GSE datasets are publicly accessible through the NCBI website (https://www.ncbi.nlm.nih.gov/). **Table S3.** RNA expression in clone OA7G vs control Panc-1 cells. The ^2^log fold change for PP2A and EMT genes was calculated for resistant clone OA7G vs untreated Panc-1 cells (FDR = false discovery rate, ns = not significant, nd = not determined). **Table S4.** Differential protein expression in Panc-1 cells exposed to Okadaic acid and clones. Protein composition of Panc-1 cells (Contr), cells exposed to OA for 24 h (OA-24) and the resistant clones OA7A and OA7G was determined. The 2log fold change (with corresponding FDR) for PP2A (A) and EMT genes (B) was calculated relative to untreated Panc-1 cells in the first block of 6 columns. In the second block, differences between the clones or between the clones and OA-24 were calculated. An FDR smaller than 0.05 is indicated in green, when FDR is between 0.05 and 0.20 this is indicated in yellow. (FDR = false discovery rate, ns = not significant, FDR > 0.20). Supplementary Material and Methods: RNA sequencing procedure.

## Data Availability

RNA sequencing data is deposited at Gene Expression Omnibus (NCBI: GSE216036). Proteomics data are available via ProteomeXchange with identifier PXD037976.

## Abbreviations

AUC: Area under the ROC Curve
CPTAC: Clinical Proteomic Tumor Analysis Consortium
ECM: Extracellular matrix
EMT: Epithelial-to-Mesenchymal transition
FDR: False discovery rate
FPKM: Fragments Per Kilobase of transcript per Million mapped reads
GSEA: Gene set enrichment analysis
HC: Hierarchical clustering
IC50: Half maximal inhibitory concentration
IPA: QIAGEN Ingenuity Pathway Analysis
meanDELTA: Mean differential gene expression (tumor/non-tumor, ^2^log)
mmu: Milli mass unit
NES: Normalized enrichment score
OA: Okadaic acid
PDAC: Pancreatic ductal adenocarcinoma
PDAssign: 62 Gene signature, Collinson et al. 2011 [5]
PP2A: Protein Phosphatase Type 2A
ppm: Parts per million
ROC: Receiver Operating Characteristic
SMAP: Small-molecule activators of PP2A
SPmean: Mean Spearman correlation
TBS: Tris-buffered saline
TCGA: The Cancer Genome Atlas
