# Supplementary material for: Human pancreatic cancer patients with Epithelial-to-Mesenchymal Transition and an aggressive phenotype show a disturbed balance in Protein Phosphatase Type 2A expression and functionality
Source: J Transl Med. 2023 May 11;21:317. doi: 10.1186/s12967-023-04145-z (PMC10176933; doi:10.1186/s12967-023-04145-z)
Supplement: Supplementary file 1 — Additional file 1: Figure S1A. Non-comprehensive overview of major growth signaling functions of PP2A complexes. A key mechanism by which PP2A exerts its tumor suppressive role resides in controlling several components downstream of RAS signaling, including PI3K/AKT, MEK/ERK and JAK/STAT, thereby preventing anomalous cell survival, growth and migration. PP2A is also more directly involved in preventing activation of diverse proto-oncogenes, including β-catenin and c-MYC, as well as in promoting activation of major tumor suppressors, such as p53 and pRb. Other established roles of PP2A occur in regulation of apoptosis, autophagy, cell division and protein synthesis. Figure S1B. Structural diversity of PP2A complexes, and their regulation by cellular inhibitors and activators. Functional PP2A complexes consist of one C and one A subunit (each encoded by two different human genes), or one C, one A and one B-type subunit (encoded by 15 different human genes, some of which give rise to multiple isoforms). Additional regulation of phosphatase activity or holoenzyme assembly occurs by endogenous cellular PP2A inhibitory proteins, such as SET, ANP32A, TIPRL, CIP2A, PME-1/PPME-1 and ARPP19, and cellular PP2A activators, such as IGBP1 and PTPA/PPP2R4. Table S1. Genes and search terms that were used to investigate the expression datasets: PP2A-search terms, PDAssign and IPA-EMT. Table S2. Spearman correlation and differentially gene expression for GSE15471, GSE16515, GSE101448 and calculated means. Legend: Table S2A. For dataset GSE15471, GSE16515 and GSE101448, the Spearman correlation (SP) and corresponding p was computed for the PP2A-genes. The mean Spearman correlation (SPmean: tumor = 1 vs non-tumor = 0) and the median for p-value was computed and expressed as 10log. Table S2B. Differential gene expression (DELTA) was also computed (tumor vs non-tumor as 2log values) and the mean was calculated (meanDELTA). A t-test was computed for the individual gens in each dataset fol [file 12967_2023_4145_MOESM1_ESM.pdf]

Supplementary data

Human pancreatic cancer patients with Epithelial-to-Mesenchymal Transition and an aggressive phenotype show a disturbed balance in Protein Phosphatase Type 2A expression and functionality

Jos van Pelt[1,2,*], Bob Meeusen[3], Rita Derua[3,4], Liesbeth Guffens[2,3], Eric Van Cutsem[1,2], Veerle Janssens[2,3,*,**] and Chris Verslype[1,2,**]

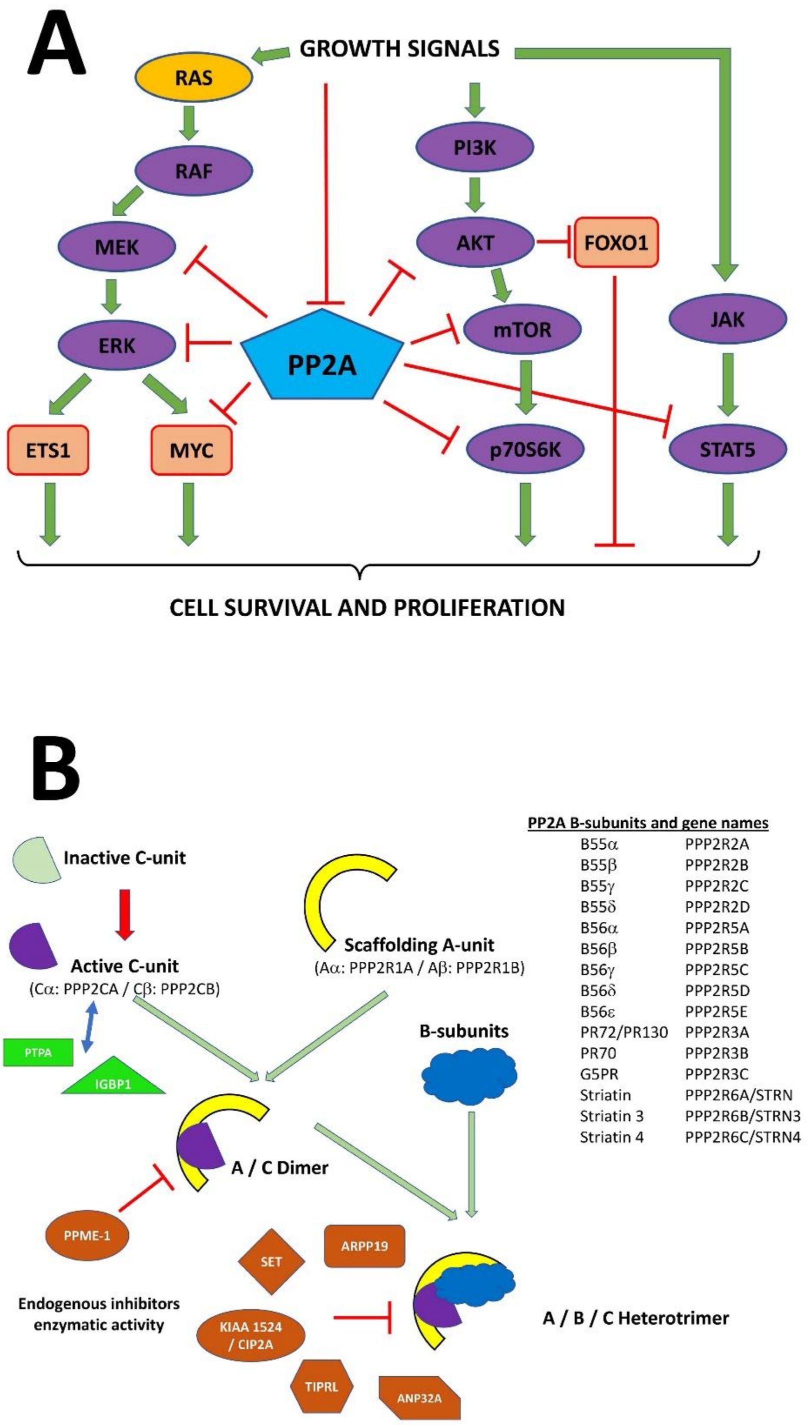

Supplementary figure S1A: Non-comprehensive overview of major growth signaling functions of PP2A complexes. A key mechanism by which PP2A exerts its tumor suppressive role resides in controlling several components downstream of RAS signaling, including PI3K/AKT, MEK/ERK and JAK/STAT, thereby preventing anomalous cell survival, growth and migration. PP2A is also more directly involved in preventing activation of diverse proto-oncogenes, including β-catenin and C-MYC, as well as in promoting activation of major tumor suppressors, such as p53 and pRb. Other established roles of PP2A occur in regulation of apoptosis, autophagy, cell division and protein synthesis [1-5]. **S1B: Structural diversity of PP2A complexes, and their regulation by cellular inhibitors and activators.** Functional PP2A complexes consist of one C and one A subunit (each encoded by two different human genes), or one C, one A and one B-type subunit (encoded by 15 different human genes, some of which give rise to multiple isoforms). Additional regulation of phosphatase activity or holoenzyme assembly occurs by endogenous cellular PP2A inhibitory proteins, such as SET, ANP32A, TIPRL, CIP2A, PME-1 and ARPP19, and cellular PP2A activators, such as IGBP1 and PTPA/PPP2R4.

Suppl table S1: Genes and search terms that were used to investigate the expression datasets

PP2A-search terms*

2PP2A	CIP2A	IPP2A2	PPP2CA	PPP2R3C	STRN
ALPHA-4	ENSA	KIAA1524	PPP2CB	PPP2R4	STRN3
ANP32a	ENSAL	LANP	PPP2R1A	PPP2R5A	STRN4
ARPP16	FAM44B	MAPM	PPP2R1B	PPP2R5B	TAF-I
ARPP-16	HPPCn	MRD58	PPP2R2A	PPP2R5C	TAF-IBETA
ARPP19	I1PP2A	PHAP1	PPP2R2B	PPP2R5D	TIP
ARPP-19	I2PP2A	PHAPI	PPP2R2C	PPP2R5E	TIP41
ARPP-19e	IBP1	PHAPII	PPP2R2D	PTPA	TIPRL
Bod1	IGAAD	PP32	PPP2R3A	SET	TIPRL1
C15orf1	IGBP1	PPME1	PPP2R3B	SETBP1	

PDAssign**

AGR2	CELA3B	FXYD3	NT5E	REG1B	ST6GALNAC1
AHNAK2	CFTR	GP2	PAPPA	REG3A	TFF1
AIM2	CKS2	GPM6B	PHLDA1	S100A2	TFF3
ATP10B	CLPS	GPRC5A	PLA2G1B	S100P	TMEM45B
CAPN8	CPB1	GPX2	PLS1	SDR16C5	TOX3
CAV1	CTRB2	HK2	PMAIP1	SLC16A1	TSPAN8
CEACAM5	ELF3	HMMR	PNLIP	SLC2A3	TWIST1
CEACAM6	ERBB3	KRT14	PNLIPRP2	SLC3A1	
CEL	FAM26F	LGALS4	PRSS1	SLC4A4	
CELA2B	FERMT1	LOX	PRSS2	SLC5A3	
CELA3A	FOXQ1	MUC13	REG1A	SPINK1	

IPA-EMT***

AKT1	FGF2	IRS1	MRAS	SNAI2	WNT3A
AKT2	FGFR1	IRS2	NOTCH1	SPP1	WNT4
BRAF	FGFR2	JAG1	NOTCH2	STAT3	WNT5A
CDH1	FOXC2	JAG2	OCLN	TGFB1	WNT11
CTNNB1	FZD7	KRAS	PIK3CA	TGFB2	ZEB1
DVL2	GSC	KRT19	PTPN11	TGFB3	ZEB2
EGF	GSK3B	LEF1	RBPJ	TGFBR1	
EGFR	HGF	MAP2K2	RHOA	TGFBR2	
EPCAM	HIF1A	mir-8	SMAD3	TJP2	
ESRP2	HMGA2	mir-155	SMAD4	TWIST1	
FGF1	HRAS	MMP2	SNAI1	VIM	

Supplemented with YAP1

*: we searched for gene names as well as synonyms
**: [7]
***: retrieved feb 2021, www.ingenuity.com

Suppl Table S2: Spearman correlation and differentially gene expression for GSE15471, GSE16515, GSE101448 and calculated means.

Legend: Table S2A: For dataset GSE15471, GSE16515 and GSE101448, the Spearman correlation (SP) and corresponding p was computed for the PP2A-genes. The mean Spearman correlation (SPmean: tumor=1 vs non-tumor=0) and the median for p-value was computed and expressed as ^{10}log. **Table S2B:** Differential gene expression (DELTA) was also computed (tumor vs non-tumor as ^{2}log values) and the mean was calculated (meanDELTA). A t-test was computed for the individual gens in each dataset followed by calculation of median p-value (^{10}log). **Table S2C:** Spearman correlation for EMT-genes. **Table S2D:** Differential gene expression for EMT genes. The in this paper used GSE datasets are publicly accessible through the NCBI website (https://www.ncbi.nlm.nih.gov/).

Table S2A

SPEARMAN CORRELATION

PP2A genes

Dataset	gse15471			gse16515			gse101448				
**GENE**	**SP**	**p (10log)**	**GENE**	**SP**	**p (10log)**	**GENE**	**SP**	**p (10log)**	**GENE**	**SPmean**	**median p (10log)**
ANP32A	0,412	-3,415	ANP32A	0,319	-1,672	ANP32A	0,328	-1,498	ANP32A	0,353	-1,672
ARPP19	0,482	-4,618	ARPP19	0,472	-3,342	ARPP19	0,442	-2,495	ARPP19	0,465	-3,342
BOD1	-0,050	-0,169	BOD1	-0,326	-1,733	BOD1	-0,215	-0,783	BOD1	-0,197	-0,783
ENSA	-0,014	-0,041	ENSA	0,375	-2,194	ENSA	-0,015	-0,035	ENSA	0,115	-0,041
IGBP1	-0,480	-4,565	IGBP1	-0,211	-0,876	IGBP1	-0,358	-1,733	IGBP1	-0,350	-1,733
KIAA1524	0,478	-4,539	KIAA1524	0,364	-2,083	KIAA1524	0,713	-10,000	KIAA1524	0,518	-4,539
PPME1	0,439	-3,833	PPME1	0,611	-5,799	PPME1	0,306	-1,334	PPME1	0,452	-3,833
PPP2CA	0,428	-3,654	PPP2CA	0,208	-0,860	PPP2CA	0,242	-0,928	PPP2CA	0,293	-0,928
PPP2CB	-0,165	-0,772	PPP2CB	0,056	-0,158	PPP2CB	0,453	-2,614	PPP2CB	0,115	-0,772
PPP2R1A	0,407	-3,331	PPP2R1A	0,303	-1,532	PPP2R1A	0,385	-1,955	PPP2R1A	0,365	-1,955
PPP2R1B	-0,601	-7,590	PPP2R1B	-0,425	-2,742	PPP2R1B	-0,781	-10,000	PPP2R1B	-0,602	-7,590
PPP2R2A	0,381	-2,951	PPP2R2A	0,278	-1,333	PPP2R2A	0,555	-3,893	PPP2R2A	0,405	-2,951
PPP2R2B	0,062	-0,217	PPP2R2B	-0,119	-0,400	PPP2R2B	-0,042	-0,102	PPP2R2B	-0,033	-0,217
PPP2R2C	-0,297	-1,917	PPP2R2C	0,056	-0,158	PPP2R2C	0,491	-3,040	PPP2R2C	0,083	-1,917
PPP2R2D	-0,388	-3,048	PPP2R2D	-0,400	-2,460	PPP2R2D	-0,853	-10,000	PPP2R2D	-0,547	-3,048
PPP2R3A	-0,045	-0,151	PPP2R3A	-0,250	-1,131	PPP2R3A	-0,317	-1,415	PPP2R3A	-0,204	-1,131
						PPP2R3B	-0,287	-1,206	PPP2R3B	-0,287	-1,206
PPP2R3C	0,283	-1,772	PPP2R3C	0,139	-0,488	PPP2R3C	0,521	-3,417	PPP2R3C	0,314	-1,772
PPP2R4	-0,008	-0,025	PPP2R4	0,305	-1,554	PPP2R4	0,174	-0,578	PPP2R4	0,157	-0,578
PPP2R5A	-0,660	-10,000	PPP2R5A	-0,064	-0,186	PPP2R5A	0,257	-1,016	PPP2R5A	-0,156	-1,016
PPP2R5B	-0,120	-0,495	PPP2R5B	0,353	-1,975	PPP2R5B	-0,026	-0,063	PPP2R5B	0,069	-0,495
PPP2R5C	0,302	-1,975	PPP2R5C	0,264	-1,230	PPP2R5C	0,049	-0,123	PPP2R5C	0,205	-1,230
PPP2R5D	-0,368	-2,780	PPP2R5D	0,017	-0,043	PPP2R5D	0,294	-1,256	PPP2R5D	-0,019	-1,256
PPP2R5E	0,599	-7,535	PPP2R5E	0,361	-2,056	PPP2R5E	0,408	-2,161	PPP2R5E	0,456	-2,161
SET	0,173	-0,830	SET	0,378	-2,223	SET	0,343	-1,613	SET	0,298	-1,613
SETBP1	-0,001	-0,004	SETBP1	-0,580	-5,121	SETBP1	-0,709	-10,000	SETBP1	-0,430	-5,121
STRN	0,437	-3,810	STRN	0,314	-1,625	STRN	0,457	-2,654	STRN	0,403	-2,654
STRN3	0,509	-5,138	STRN3	0,253	-1,151	STRN3	0,404	-2,126	STRN3	0,389	-2,126
STRN4	0,228	-1,254	STRN4	0,311	-1,600	STRN4	0,275	-1,133	STRN4	0,271	-1,254
TIPRL	0,469	-4,361	TIPRL	0,441	-2,943	TIPRL	0,313	-1,387	TIPRL	0,408	-2,943

Table S2B

DIFFERENTIAL GENE EXPRESSION

PP2A genes

Dataset	gse15471			gse16515			gse101448				
**GENE**	**DELTA**	**p (10log)**	**GENE**	**DELTA**	**p (10log)**	**GENE**	**DELTA**	**p (10log)**	**GENE**	**meanDELTA**	**median p (10log)**
ANP32A	0,362	-4,286	ANP32A	0,290	-2,219	ANP32A	0,484	-1,791	ANP32A	0,379	-2,219
ARPP19	0,351	-4,644	ARPP19	0,379	-3,443	ARPP19	0,519	-1,918	ARPP-19	0,416	-3,443
BOD1	-0,046	-0,299	BOD1	-0,282	-1,490	BOD1	-0,324	-0,810	BOD1	-0,217	-0,810
ENSA	0,002	-0,017	ENSA	0,339	-2,244	ENSA	-0,020	-0,119	ENSA	0,107	-0,119
IGBP1	-0,275	-4,356	IGBP1	-0,246	-1,132	IGBP1	-0,362	-2,024	IGBP1	-0,294	-2,024
KIAA1524	0,251	-3,868	KIAA1524	0,319	-1,683	KIAA1524	0,389	-5,219	KIAA1524	0,320	-3,868
PPME1	0,153	-3,971	PPME1	0,416	-5,808	PPME1	0,267	-1,263	PPME1	0,279	-3,971
PPP2CA	0,243	-2,826	PPP2CA	0,116	-0,705	PPP2CA	0,229	-0,818	PPP2CA	0,196	-0,818
PPP2CB	-0,035	-0,259	PPP2CB	0,046	-0,214	PPP2CB	0,315	-2,334	PPP2CB	0,109	-0,259
PPP2R1A	0,190	-2,995	PPP2R1A	0,304	-1,354	PPP2R1A	0,748	-1,730	PPP2R1A	0,414	-1,730
PPP2R1B	-0,449	-7,082	PPP2R1B	-0,443	-2,995	PPP2R1B	-1,020	-9,443	PPP2R1B	-0,637	-7,082
PPP2R2A	0,241	-3,950	PPP2R2A	0,218	-1,506	PPP2R2A	0,367	-2,163	PPP2R2A	0,275	-2,163
PPP2R2B	-0,002	-0,016	PPP2R2B	-0,098	-0,549	PPP2R2B	-0,052	-0,178	PPP2R2B	-0,051	-0,178
PPP2R2C	-0,163	-1,898	PPP2R2C	0,104	-0,432	PPP2R2C	0,671	-3,437	PPP2R2C	0,204	-1,898
PPP2R2D	-0,562	-3,801	PPP2R2D	-1,083	-4,393	PPP2R2D	-1,524	-11,705	PPP2R2D	-1,056	-4,393
PPP2R3A	0,010	-0,035	PPP2R3A	-0,179	-0,611	PPP2R3A	-0,217	-1,691	PPP2R3A	-0,129	-0,611
						PPP2R3B	-0,433	-1,229	PPP2R3B	-0,433	-1,229
PPP2R3C	0,320	-3,026	PPP2R3C	0,138	-0,775	PPP2R3C	0,746	-1,819	PPP2R3C	0,401	-1,819
PPP2R4	0,010	-0,085	PPP2R4	0,235	-1,123	PPP2R4	0,235	-0,824	PPP2R4	0,160	-0,824
PPP2R5A	-0,398	-9,301	PPP2R5A	-0,047	-0,276	PPP2R5A	0,589	-0,941	PPP2R5A	0,048	-0,941
PPP2R5B	-0,045	-0,468	PPP2R5B	0,207	-1,443	PPP2R5B	0,194	-0,268	PPP2R5B	0,119	-0,468
PPP2R5C	0,162	-1,143	PPP2R5C	0,200	-1,252	PPP2R5C	0,071	-0,315	PPP2R5C	0,144	-1,143
PPP2R5D	-0,132	-2,100	PPP2R5D	0,014	-0,050	PPP2R5D	0,288	-1,652	PPP2R5D	0,057	-1,652
PPP2R5E	0,514	-8,408	PPP2R5E	0,389	-3,149	PPP2R5E	0,457	-1,051	PPP2R5E	0,453	-3,149
SET	0,116	-0,931	SET	0,396	-2,494	SET	0,504	-0,638	SET	0,339	-0,931
SETBP1	-0,003	-0,007	SETBP1	-1,021	-4,327	SETBP1	-0,932	-4,530	SETBP1	-0,652	-4,327
STRN	0,359	-5,193	STRN	0,214	-1,914	STRN	0,290	-0,942	STRN	0,288	-1,914
STRN3	0,346	-4,640	STRN3	0,221	-1,276	STRN3	0,399	-2,136	STRN3	0,322	-2,136
STRN4	0,128	-1,229	STRN4	0,282	-1,118	STRN4	0,190	-0,548	STRN4	0,200	-1,118
TIPRL	0,373	-4,978	TIPRL	0,406	-2,898	TIPRL	0,163	-0,619	TIPRL	0,314	-2,898

Table S2C

SPEARMAN CORRELATION

EMT genes

Dataset	gse15471			gse16515			gse101448				
**GENE**	**SP**	**p (10log)**	**GENE**	**SP**	**p (10log)**	**GENE**	**SP**	**p (10log)**	**GENE**	**SPmean**	**median p (10log)**
AKT1	0,180	-0,876	AKT1	0,397	-2,428	AKT1	-0,072	-0,190	AKT1	0,168	-0,876
AKT2	-0,305	-2,007	AKT2	0,028	-0,074	AKT2	-0,302	-1,308	AKT2	-0,193	-1,308
BRAF	0,195	-0,987	BRAF	0,089	-0,277	BRAF	-0,468	-2,780	BRAF	-0,061	-0,987
CDH1	-0,030	-0,096	CDH1	0,478	-3,418	CDH1	0,068	-0,178	CDH1	0,172	-0,178
CTNNB1	0,580	-6,879	CTNNB1	0,511	-3,903	CTNNB1	0,170	-0,561	CTNNB1	0,420	-3,903
DVL2	0,280	-1,745	DVL2	0,308	-1,578	DVL2	0,223	-0,824	DVL2	0,270	-1,578
EGF	-0,430	-3,697	EGF	-0,389	-2,339	EGF	-0,853	-10,000	EGF	-0,557	-3,697
EGFR	-0,216	-1,151	EGFR	-0,100	-0,320	EGFR	-0,204	-0,724	EGFR	-0,173	-0,724
EPCAM	0,077	-0,282	EPCAM	0,514	-3,947	EPCAM	0,211	-0,762	EPCAM	0,267	-0,762
						ESRP2	-0,577	-4,244	ESRP2	-0,577	-4,244
FGF1	0,426	-3,631	FGF1	0,314	-1,625	FGF1	0,577	-4,244	FGF1	0,439	-3,631
FGF2	0,392	-3,107	FGF2	-0,017	-0,043	FGF2	0,438	-2,457	FGF2	0,271	-2,457
FGFR1	-0,345	-2,478	FGFR1	-0,580	-5,121	FGFR1	-0,811	-10,000	FGFR1	-0,579	-5,121
FGFR2	-0,323	-2,210	FGFR2	-0,303	-1,532	FGFR2	-0,713	-10,000	FGFR2	-0,446	-2,210
FOXC2	0,263	-1,567	FOXC2	0,383	-2,280	FOXC2	0,755	-10,000	FOXC2	0,467	-2,280
FZD7	0,526	-5,523	FZD7	0,258	-1,190	FZD7	0,566	-4,064	FZD7	0,450	-4,064
GSC	-0,399	-3,208	GSC	-0,167	-0,627	GSC	-0,234	-0,886	GSC	-0,267	-0,886
GSK3B	0,570	-9,827	GSK3B	0,464	-3,231	GSK3B	0,477	-2,886	GSK3B	0,504	-3,231
HGF	0,548	-6,035	HGF	-0,150	-0,542	HGF	0,279	-1,157	HGF	0,226	-1,157
HIF1A	0,614	-8,423	HIF1A	0,219	-0,928	HIF1A	0,702	-10,000	HIF1A	0,512	-8,423
HMGA2	0,054	-0,183	HMGA2	0,130	-0,450	HMGA2	0,525	-3,467	HMGA2	0,236	-0,450
HRAS	0,385	-3,009	HRAS	0,369	-2,138	HRAS	0,049	-0,123	HRAS	0,268	-2,138
IRS1	0,777	-10,000	IRS1	0,475	-3,380	IRS1	0,653	-5,842	IRS1	0,635	-5,842
IRS2	0,466	-4,311	IRS2	0,047	-0,131	IRS2	0,415	-2,233	IRS2	0,309	-2,233
JAG1	0,430	-3,697	JAG1	0,405	-2,521	JAG1	0,113	-0,330	JAG1	0,316	-2,521
JAG2	0,085	-0,320	JAG2	0,236	-1,037	JAG2	-0,015	-0,035	JAG2	0,102	-0,320
KRAS	0,417	-3,479	KRAS	0,019	-0,051	KRAS	0,642	-5,520	KRAS	0,359	-3,479
KRT19	0,654	-10,000	KRT19	0,755	-10,000	KRT19	0,740	-10,000	KRT19	0,716	-10,000
LEF1	0,577	-6,799	LEF1	0,461	-3,193	LEF1	0,657	-5,963	LEF1	0,565	-5,963
MAP2K2	-0,443	-3,903	MAP2K2	0,008	-0,021	MAP2K2	-0,238	-0,907	MAP2K2	-0,224	-0,907
MMP2	0,661	-10,000	MMP2	0,139	-0,488	MMP2	0,657	-5,963	MMP2	0,486	-5,963
MRAS	0,573	-9,967	MRAS	0,105	-0,342	MRAS	0,309	-1,361	MRAS	0,329	-1,361
NOTCH1	-0,373	-2,836	NOTCH1	-0,275	-1,312	NOTCH1	-0,464	-2,738	NOTCH1	-0,371	-2,738
NOTCH2	0,539	-5,807	NOTCH2	0,033	-0,089	NOTCH2	-0,177	-0,595	NOTCH2	0,132	-0,595
OCLN	0,052	-0,178	OCLN	0,261	-1,210	OCLN	-0,325	-1,470	OCLN	-0,004	-1,210
PIK3CA	0,500	-4,971	PIK3CA	0,092	-0,287	PIK3CA	0,279	-1,157	PIK3CA	0,290	-1,157
PTPN11	0,088	-0,333	PTPN11	-0,122	-0,412	PTPN11	0,170	-0,561	PTPN11	0,045	-0,412
RBPJ	0,412	-3,415	RBPJ	0,075	-0,225	RBPJ	0,366	-1,796	RBPJ	0,284	-1,796
RHOA	0,461	-4,212	RHOA	0,383	-2,280	RHOA	0,298	-1,282	RHOA	0,381	-2,280
SMAD3	0,466	-4,311	SMAD3	0,630	-6,327	SMAD3	0,177	-0,595	SMAD3	0,424	-4,311
SMAD4	-0,168	-0,790	SMAD4	-0,652	-7,266	SMAD4	-0,464	-2,738	SMAD4	-0,428	-2,738
SNAI1	0,034	-0,111	SNAI1	-0,341	-1,870	SNAI1	0,068	-0,178	SNAI1	-0,080	-0,178
SNAI2	0,603	-7,712	SNAI2	0,125	-0,425	SNAI2	0,642	-5,520	SNAI2	0,457	-5,520
SPP1	0,603	-7,712	SPP1	0,189	-0,747	SPP1	0,283	-1,182	SPP1	0,358	-1,182
STAT3	0,507	-5,110	STAT3	0,186	-0,730	STAT3	0,309	-1,361	STAT3	0,334	-1,361
TGFB1	0,495	-4,860	TGFB1	0,372	-2,166	TGFB1	0,472	-2,821	TGFB1	0,446	-2,821
TGFB2	0,569	-9,767	TGFB2	0,375	-2,194	TGFB2	0,223	-0,824	TGFB2	0,389	-2,194
TGFB3	0,378	-2,914	TGFB3	0,006	-0,014	TGFB3	0,143	-0,447	TGFB3	0,176	-0,447
TGFBR1	0,638	-10,000	TGFBR1	0,391	-2,369	TGFBR1	0,313	-1,387	TGFBR1	0,447	-2,369
TGFBR2	0,418	-3,500	TGFBR2	0,114	-0,377	TGFBR2	0,340	-1,583	TGFBR2	0,291	-1,583
TJP2	0,254	-1,488	TJP2	0,305	-1,554	TJP2	0,230	-0,863	TJP2	0,263	-1,488
TWIST1	0,654	-10,000	TWIST1	0,450	-3,050	TWIST1	0,740	-10,000	TWIST1	0,615	-10,000
VIM	0,485	-4,670	VIM	0,019	-0,051	VIM	0,170	-0,561	VIM	0,225	-0,561
WNT11	-0,399	-3,208	WNT11	-0,239	-1,056	WNT11	-0,317	-1,415	WNT11	-0,318	-1,415
						WNT3A	0,019	-0,044	WNT3A	0,019	-0,044
WNT4	0,011	-0,033	WNT4	-0,200	-0,810	WNT4	-0,019	-0,044	WNT4	-0,069	-0,044
WNT5A	0,661	-10,000	WNT5A	0,552	-4,593	WNT5A	0,245	-0,951	WNT5A	0,486	-4,593
YAP1	0,694	-10,000	YAP1	0,589	-5,294	YAP1	0,381	-1,924	YAP1	0,555	-5,294
						ZEB1	0,623	-5,080	ZEB1	0,623	-5,080
ZEB2	0,459	-4,187	ZEB2	0,039	-0,106	ZEB2	0,408	-2,161	ZEB2	0,302	-2,161

Table S2D

DIFFERENTIAL GENE EXPRESSION

EMT genes

Dataset	gse15471			gse16515			gse101448				
**GENE**	**DELTA**	**p (10log)**	**GENE**	**DELTA**	**p (10log)**	**GENE**	**DELTA**	**p (10log)**	**GENE**	**meanDELTA**	**median p (10log)**
AKT1	0,113	-1,296	AKT1	0,291	-1,510	AKT1	-0,006	-0,031	AKT1	0,132	-1,296
AKT2	-0,119	-1,938	AKT2	0,026	-0,084	AKT2	-0,479	-0,989	AKT2	-0,191	-0,989
BRAF	0,104	-0,994	BRAF	0,098	-0,632	BRAF	-0,460	-2,912	BRAF	-0,086	-0,994
CDH1	0,154	-0,257	CDH1	0,956	-3,451	CDH1	0,124	-0,148	CDH1	0,411	-0,257
CTNNB1	0,620	-7,784	CTNNB1	0,516	-4,582	CTNNB1	0,080	-0,624	CTNNB1	0,405	-4,582
DVL2	0,102	-1,698	DVL2	0,214	-1,838	DVL2	0,483	-1,431	DVL2	0,266	-1,698
EGF	-2,226	-4,187	EGF	-2,820	-4,084	EGF	-2,941	-18,676	EGF	-2,663	-4,187
EGFR	-0,100	-1,025	EGFR	-0,034	-0,187	EGFR	-0,176	-0,786	EGFR	-0,103	-0,786
EPCAM	0,751	-0,925	EPCAM	0,808	-3,044	EPCAM	0,148	-0,197	EPCAM	0,569	-0,925
						ESRP2	-0,823	-2,893	ESRP2	-0,823	-2,893
FGF1	0,281	-3,080	FGF1	0,391	-1,658	FGF1	0,305	-2,854	FGF1	0,326	-2,854
FGF2	0,337	-1,615	FGF2	0,066	-0,095	FGF2	0,231	-1,546	FGF2	0,211	-1,546
FGFR1	-0,366	-2,035	FGFR1	-0,851	-6,158	FGFR1	-0,757	-8,039	FGFR1	-0,658	-6,158
FGFR2	-0,250	-1,656	FGFR2	-0,236	-1,199	FGFR2	-1,639	-5,924	FGFR2	-0,709	-1,656
FOXC2	0,290	-1,693	FOXC2	0,423	-2,117	FOXC2	1,468	-6,687	FOXC2	0,727	-2,117
FZD7	1,287	-6,549	FZD7	0,765	-1,750	FZD7	0,795	-4,414	FZD7	0,949	-4,414
GSC	-0,237	-3,652	GSC	0,032	-0,272	GSC	-0,062	-0,824	GSC	-0,089	-0,824
GSK3B	0,280	-7,205	GSK3B	0,242	-3,304	GSK3B	0,340	-1,647	GSK3B	0,287	-3,304
HGF	0,539	-5,284	HGF	-0,199	-0,658	HGF	0,302	-1,162	HGF	0,214	-1,162
HIF1A	1,017	-10,000	HIF1A	0,462	-1,922	HIF1A	0,546	-7,010	HIF1A	0,675	-7,010
HMGA2	0,111	-1,111	HMGA2	0,074	-0,472	HMGA2	0,750	-2,606	HMGA2	0,312	-1,111
HRAS	0,307	-3,414	HRAS	0,404	-1,621	HRAS	0,058	-0,308	HRAS	0,256	-1,621
IRS1	1,126	-13,149	IRS1	0,661	-3,354	IRS1	1,328	-4,512	IRS1	1,038	-4,512
IRS2	0,543	-4,549	IRS2	0,104	-0,239	IRS2	0,213	-2,103	IRS2	0,287	-2,103
JAG1	0,292	-3,771	JAG1	0,324	-1,880	JAG1	0,208	-0,249	JAG1	0,274	-1,880
JAG2	0,155	-1,082	JAG2	0,248	-0,872	JAG2	0,037	-0,058	JAG2	0,147	-0,872
KRAS	0,192	-3,705	KRAS	0,034	-0,152	KRAS	0,584	-3,535	KRAS	0,270	-3,535
KRT19	1,697	-9,064	KRT19	2,418	-10,529	KRT19	2,224	-8,134	KRT19	2,113	-9,064
LEF1	0,872	-7,374	LEF1	0,887	-4,361	LEF1	1,755	-6,648	LEF1	1,172	-6,648
MAP2K2	-0,226	-4,885	MAP2K2	0,021	-0,069	MAP2K2	-0,103	-0,408	MAP2K2	-0,103	-0,408
MMP2	0,675	-11,136	MMP2	0,153	-0,731	MMP2	1,657	-4,946	MMP2	0,828	-4,946
MRAS	0,416	-5,965	MRAS	0,149	-0,483	MRAS	0,443	-0,860	MRAS	0,336	-0,860
NOTCH1	-0,127	-1,415	NOTCH1	-0,136	-1,329	NOTCH1	-0,437	-1,873	NOTCH1	-0,234	-1,415
NOTCH2	0,648	-6,889	NOTCH2	0,094	-0,355	NOTCH2	-0,106	-0,573	NOTCH2	0,212	-0,573
OCLN	0,070	-0,145	OCLN	0,496	-1,620	OCLN	-0,138	-1,453	OCLN	0,143	-1,453
PIK3CA	0,501	-5,604	PIK3CA	0,068	-0,289	PIK3CA	0,217	-0,833	PIK3CA	0,262	-0,833
PTPN11	0,012	-0,114	PTPN11	-0,042	-0,270	PTPN11	0,120	-0,567	PTPN11	0,030	-0,270
RBPJ	0,272	-3,186	RBPJ	0,121	-0,458	RBPJ	0,234	-1,136	RBPJ	0,209	-1,136
RHOA	0,254	-5,154	RHOA	0,290	-2,553	RHOA	0,428	-0,950	RHOA	0,324	-2,553
SMAD3	0,286	-3,449	SMAD3	0,743	-5,152	SMAD3	0,193	-0,531	SMAD3	0,407	-3,449
SMAD4	-0,084	-1,061	SMAD4	-0,408	-6,491	SMAD4	-0,598	-1,264	SMAD4	-0,363	-1,264
SNAI1	0,055	-0,264	SNAI1	-0,308	-1,233	SNAI1	0,032	-0,344	SNAI1	-0,074	-0,344
SNAI2	1,692	-8,945	SNAI2	0,653	-1,120	SNAI2	0,987	-3,108	SNAI2	1,111	-3,108
SPP1	2,131	-8,539	SPP1	0,702	-1,140	SPP1	0,356	-1,103	SPP1	1,063	-1,140
STAT3	0,388	-5,616	STAT3	0,168	-0,814	STAT3	0,227	-0,739	STAT3	0,261	-0,814
TGFB1	0,487	-6,114	TGFB1	0,628	-2,870	TGFB1	0,740	-1,614	TGFB1	0,618	-2,870
TGFB2	0,636	-5,816	TGFB2	0,432	-1,900	TGFB2	0,438	-0,633	TGFB2	0,502	-1,900
TGFB3	0,245	-2,617	TGFB3	0,018	-0,042	TGFB3	0,241	-0,213	TGFB3	0,168	-0,213
TGFBR1	1,068	-9,679	TGFBR1	0,748	-3,445	TGFBR1	0,299	-0,974	TGFBR1	0,705	-3,445
TGFBR2	0,443	-2,722	TGFBR2	0,068	-0,125	TGFBR2	0,402	-1,047	TGFBR2	0,304	-1,047
TJP2	0,172	-1,321	TJP2	0,323	-1,990	TJP2	0,141	-0,429	TJP2	0,212	-1,321
TWIST1	1,688	-7,562	TWIST1	1,234	-3,120	TWIST1	2,051	-8,012	TWIST1	1,658	-7,562
VIM	0,999	-4,631	VIM	0,022	-0,038	VIM	0,097	-0,424	VIM	0,373	-0,424
WNT11	-0,352	-3,278	WNT11	-0,238	-0,517	WNT11	-0,648	-1,421	WNT11	-0,413	-1,421
						WNT3A	-0,062	-0,238	WNT3A	-0,062	-0,238
WNT4	0,022	-0,111	WNT4	-0,214	-0,844	WNT4	0,020	-0,029	WNT4	-0,057	-0,111
WNT5A	1,195	-8,696	WNT5A	1,455	-4,214	WNT5A	0,493	-0,870	WNT5A	1,048	-4,214
YAP1	0,966	-11,317	YAP1	0,808	-5,571	YAP1	0,525	-1,323	YAP1	0,766	-5,571
						ZEB1	1,188	-4,194	ZEB1	1,188	-4,194
ZEB2	0,725	-4,413	ZEB2	0,082	-0,209	ZEB2	0,540	-2,472	ZEB2	0,449	-2,472

Suppl Table S3: RNA expression in clone OA7G vs control Panc-1 cells. The ^{2}log fold change for PP2A and EMT genes was calculated for resistant clone OA7G vs untreated Panc-1 cells (FDR = false discovery rate, ns = not significant, nd = not determined).

PP2A genes

GENE	OA7G-Con	FDR
ANP32A	0.97	4.75E-05
ARPP19	-0.66	2.71E-03
BOD1	-0.67	1.57E-03
ENSA	-0.17	ns
IGBP1	0.19	4.29E-03
KIAA1524	-0.58	ns
PPME1	-0.28	4.65E-03
PPP2CA	-0.59	1.35E-03
PPP2CB	0.13	ns
PPP2R1A	0.32	8.02E-03
PPP2R1B	-0.30	3.52E-04
PPP2R2A	0.22	ns
PPP2R2B	-6.46	3.69E-04
PPP2R2C	0.02	ns
PPP2R2D	-0.57	1.36E-03
PPP2R3A	1.01	1.19E-02
PPP2R3B	-0.91	3.29E-03
PPP2R3C	0.59	9.55E-03
PPP2R5A	0.03	ns
PPP2R5B	0.25	ns
PPP2R5C	0.86	6.81E-04
PPP2R5D	-0.61	9.90E-05
PPP2R5E	0.81	6.77E-04
PTPA	0.00	ns
SET	-0.07	2.14E-02
SETBP1	2.21	2.04E-06
STRN	0.23	7.74E-03
STRN3	1.19	1.58E-04
STRN4	0.35	2.71E-03
TIPRL	-0.68	4.73E-04

EMT genes

GENE	OA7G-Con	FDR	GENE	OA7G-Con	FDR
AKT1	0.51	6.23E-03	*MAP2K2*	0.31	3.50E-04
AKT2	0.95	1.71E-02	*MMP2*	0.12	ns
BRAF	1.00	3.00E-04	*MRAS*	0.34	ns
CDH1	0.16	ns	*NOTCH1*	0.51	2.20E-02
CTNNB1	0.65	9.52E-04	*NOTCH2*	0.32	ns
DVL2	-0.71	1.88E-02	*OCLN*	-0.70	ns
EGF	nd		*PIK3CA*	0.18	ns
EGFR	-0.08	ns	*PTPN11*	0.06	ns
EpCAM	1.94	5.09E-04	*RBPJ*	0.24	ns
ESRP2	-0.78	2.60E-03	*RHOA*	-0.02	ns
FGF1	3.73	4.02E-02	*SMAD3*	0.70	1.35E-02
FGF2	-2.27	2.73E-04	*SMAD4*	1.08	3.12E-04
FGFR1	1.11	4.22E-03	*SNAI1*	0.63	ns
FGFR2	-5.42	7.81E-04	*SNAI2*	0.44	ns
FOXC2	0.28	ns	*STAT3*	-0.35	ns
FZD7	-3.26	1.10E-04	*TGFB1*	1.60	1.48E-03
GSC	nd		*TGFB2*	-1.38	2.51E-02
GSK3B	-0.29	ns	*TGFB3*	1.53	3.41E-03
HGF	-0.25	ns	*TGFBR1*	1.28	3.29E-03
HIF1A	0.70	1.12E-03	*TGFBR2*	1.07	2.00E-04
HMGA2	0.78	ns	*TJP2*	-0.65	ns
HRAS	-0.44	ns	*TWIST1*	nd	
IRS1	-0.58	4.47E-03	*VIM*	0.35	ns
IRS2	1.67	2.34E-04	*WNT3A*	nd	
ITGA5	0.69	2.55E-02	*WNT4*	0.68	ns
JAG1	2.11	1.35E-03	*WNT5A*	0.92	ns
JAG2	1.27	4.00E-04	*WNT11*	4.86	5.54E-04
KRAS	0.84	5.80E-04	*YAP1*	-0.03	ns
KRT19	-0.37	1.24E-03	*ZEB1*	-0.65	2.77E-03
LEF1	-3.50	ns	*ZEB2*	6.73	4.57E-03

Suppl table S4: Differential protein expression in Panc-1 cells exposed to Okadaic acid and clones. Protein composition of Panc-1 cells (Contr), cells exposed to OA for 24 hours (OA-24) and the resistant clones OA7A and OA7G was determined. The ^{2}log fold change (with corresponding FDR) for PP2A (**A**) and EMT genes (**B**) was calculated relative to untreated Panc-1 cells in the first block of 6 columns. In the second block, differences between the clones or between the clones and OA-24 was calculated. An FDR smaller than 0.05 is indicated in green, when FDR is between 0.05 and 0.20 this is indicated in yellow. (FDR = false discovery rate, ns = not significant, FDR >0.20).

A

	DELTA tov CONTR											
**GENE**	**OA-24**	**FDR**	**OA7A**	**FDR**	**OA7G**	**FDR**	**OA7G-OA7A**	**FDR**	**OA7G-OA-24**	**FDR**	**OA7A-OA-24**	**FDR**
ARPP19	0.003	ns	0.140	ns	1.524	0.036	1.384	0.126	1.521	0.070	0.137	ns
ENSA	0.866	ns	0.745	ns	2.050	0.056	1.305	ns	1.185	0.176	-0.121	ns
IGBP1	0.165	ns	0.461	0.162	0.563	0.024	0.102	ns	0.398	0.125	0.296	ns
PPME1	0.302	ns	0.253	ns	0.477	ns	0.224	ns	0.174	ns	-0.049	ns
PPP2CA	-0.184	ns	-0.363	ns	-0.153	ns	0.211	ns	0.031	ns	-0.179	ns
PPP2R1A	0.289	ns	0.623	0.090	1.128	0.029	0.505	0.123	0.839	0.151	0.334	ns
PPP2R1B	0.868	ns	0.594	ns	0.824	0.044	0.421	ns	1.069	0.035	0.648	0.070
PPP2R2A	0.697	ns	0.381	ns	0.348	ns	-0.033	ns	-0.348	ns	-0.315	ns
PPP2RC5	0.077	ns	0.852	ns	0.572	ns	-0.279	ns	0.495	ns	0.774	ns
PPP2R5D	0.278	ns	0.262	ns	0.198	ns	-0.064	ns	-0.080	ns	-0.015	ns
PPP2R5E	-0.012	ns	1.116	0.099	1.239	0.048	0.123	ns	1.251	0.068	1.128	0.135
PTPA	0.840	ns	0.639	ns	1.208	0.069	0.569	ns	0.368	ns	-0.201	ns
SET	0.251	ns	0.597	ns	0.168	ns	-0.429	ns	-0.082	ns	0.346	ns
STRN	0.207	ns	0.389	ns	0.811	0.044	0.421	ns	0.603	0.107	0.182	ns
STRN3	0.062	ns	0.670	0.193	0.811	0.065	0.140	ns	0.749	0.117	0.608	ns
TIPRL	0.181	ns	0.201	ns	0.827	0.092	0.626	0.159	0.646	0.188	0.020	ns

B

	DELTA tov CONTR											
**GENE**	**OA-24**	**FDR**	**OA7A**	**FDR**	**OA7G**	**FDR**	**OA7G-OA7A**	**FDR**	**OA7G-OA-24**	**FDR**	**OA7A-OA-24**	**FDR**
AKT2	0.026	ns	0.569	ns	0.718	ns	0.149	ns	0.692	ns	0.543	ns
CTNNB1	0.071	ns	-1.817	0.071	-3.545	0.018	-1.728	0.097	-3.615	0.013	-1.887	0.035
EGFR	0.005	ns	-0.310	ns	-0.734	0.196	-0.424	ns	-0.738	0.153	-0.315	ns
EPCAM	0.194	ns	1.237	ns	1.019	ns	-0.218	ns	0.825	ns	1.043	ns
HMGA2	0.121	ns	0.498	ns	0.618	ns	0.119	ns	0.496	0.185	0.377	ns
HRAS	0.026	ns	0.167	ns	-0.660	0.190	-0.826	ns	-0.686	ns	0.141	ns
KRAS	0.058	ns	-0.327	ns	-0.528	ns	-0.201	ns	-0.586	ns	-0.385	ns
KRT19	0.667	ns	-0.046	ns	-1.649	0.168	-1.601	0.093	-2.224	0.077	-0.758	ns
MAP2K2	0.398	ns	0.801	0.142	1.107	0.045	0.306	ns	0.709	ns	0.403	ns
OCLN	-0.419	ns	-0.751	ns	-0.910	0.044	-0.159	ns	-0.491	ns	-0.331	ns
PTPN11	0.245	ns	0.927	0.081	1.282	0.020	0.356	ns	1.038	0.035	0.682	ns
RHOA	-0.456	ns	-0.026	ns	-0.582	ns	-0.556	ns	-0.126	ns	0.431	ns
SMAD3	0.015	ns	1.395	ns	-0.147	ns	-1.542	0.129	-0.162	ns	1.380	0.067
SMAD4	0.758	ns	1.366	ns	1.032	ns	-0.334	ns	0.274	ns	0.608	ns
STAT3	0.270	ns	-0.102	ns	-0.089	ns	0.013	ns	-0.359	ns	-0.372	ns
VIM	-0.212	ns	-0.080	ns	0.510	0.093	0.590	0.143	0.722	0.136	0.132	ns
YAP1	-0.312	ns	-0.075	ns	0.658	0.097	0.733	ns	0.970	0.079	0.237	ns

Supplementary Material and Methods: RNA sequencing procedure

Laboratory procedures

RNA quality control

RNA concentration and purity were determined spectophotometrically using the Nanodrop ND8000 (Nanodrop Technologies) and RNA integrity was assessed using a Bioanalyzer 2100 (Agilent).

Library preparation

Per sample, an amount of 500 ng of total RNA was used as input. Using the Illumina TruSeq® Stranded mRNA Sample Prep Kit (protocol version: Part # 1000000040498 v00 - October 2017) poly-A containing mRNA molecules were purified from the total RNA input using poly-T oligo-attached magnetic beads. In a reverse transcription reaction using random primers, RNA was converted into first strand cDNA and subsequently converted into double-stranded cDNA in a second strand cDNA synthesis reaction using DNA Polymerase I and RNAse H. The cDNA fragments were extended with a single 'A' base to the 3' ends of the blunt-ended cDNA fragments after which multiple indexing adapters were ligated introducing different barcodes for each sample. Finally, enrichment PCR was carried out to enrich those DNA fragments that have adapter molecules on both ends and to amplify the amount of DNA in the library.

Sequencing

Sequence-libraries of each sample were equimolarly pooled and sequenced on Illumina NovaSeq 6000 (100 bp, Single Reads, v1.5) at the VIB Nucleomics Core (www.nucleomics.be).

Data analysis

Preprocessing

Low quality ends and adapter sequences were trimmed off from the Illumina reads with FastX 0.0.14 and Cutadapt 1.15 [8,9]. Subsequently, small reads (length < 35 bp), polyA-reads (more than 90 % of the bases equal A), ambiguous reads (containing N), low-quality reads (more than 50 % of the bases < Q25) and artifact reads (all but three bases in the read equal one base type) were filtered using FastX 0.0.14 and ShortRead 1.40.0 [10]. With Bowtie2 2.3.3.1 we identified and removed reads that align to phix_illumina [11].

Mapping

The preprocessed reads were aligned with STAR aligner v2.5.2b to the reference genome of Homo sapiens (GRCh38) [12]. Default STAR aligner parameter settings were used, except for '--outSAMprimaryFlag OneBestScore --twopassMode Basic --alignIntronMin 50 --alignIntronMax 500000 --outSAMtype BAM SortedByCoordinate'. Using Samtools 1.5, reads with a mapping quality smaller than 20 were removed from the alignments [13].

Counting

The number of reads in the alignments that overlap with gene features were counted with featureCounts 1.5.3 [14]. Following parameters were chosen: -Q 0 -s 2 -t exon -g gene_id. We removed genes for which all samples had less than 1 count-per-million. Raw counts were further corrected within samples for GC-content and between samples using full quantile normalization, as implemented in the EDASeq package from Bioconductor [15].

References

1 Eichhorn, P.J.A.; Creyghton, M.P.; Bernards, R. Protein phosphatase 2A regulatory subunits and cancer. *Biochim. Biophys. Acta – Rev. Cancer* **2009**, 1795(1): 1-15. https://doi.org 10.1016/j.bbcan.2008.05.005.

2 Mazhar, S.; Taylor, S.E.; Sangodkar, J.; Narla, G. Targeting PP2A in cancer: Combination therapies. *Biochim. Biophys. Acta-Mol. Cell Res.* **2019**, 1866(1): 51-63. https://doi.org 10.1016/j.bbamcr.2018.08.020.

3 Janssens, V.; Rebollo, A. The role and therapeutic potential of Ser/Thr phosphatase PP2A in apoptotic signaling networks in human cancer cells. *Curr. Mol. Med.* **2012**, 12(3): 268-287. https://doi.org 10.2174/156652412799218930.

4 Meeusen, B.; Janssens, V. Tumor suppressive protein phosphatases in human cancer: Emerging targets for therapeutic intervention and tumor stratification. *Int. J. Biochem. Cell Biol.* **2018**, 96: 98-134. https://doi.org 10.1016/j.biocel.2017.10.002.

5 Lambrecht, C.; Haesen, D.; Sents, W.; et al. Structure regulation and pharmacological modulation of PP2A phosphatases. *Methods Mol. Biol.* **2013**, 1053: 283-305. https://doi.org 10.1007/978-1-62703-562-0_17.

6 Janky, R.; Binda, M.M.; Allemeersch, J.; et al. Prognostic relevance of molecular subtypes and master regulators in pancreatic ductal adenocarcinoma. *BMC Cancer* **2016**, 16: 632. https://doi.org 10.1186/s12885-016-2540-6.

7 Collisson, E.A.; Sadanandam, A.; Olson, P.; et al. Subtypes of pancreatic ductal adenocarcinoma and their differing responses to therapy. *Nat. Med.* **2011**, 17: 500–503. https://doi.org 10.1038/nm.2344.

8 HannonLab. Fastx-toolkit. http://hannonlab.cshl.edu/fastx_toolkit/index.html, 2010

9 Martin, M. Cutadapt removes adapter sequences from high-throughput sequencing reads. *EMBnet.journal*. **2011**, 17(1). 10-12, doi:https://doi.org/10.14806/ej.17.1.200.

10 Morgan, M.; Anders, S.; Lawrence, M.; Aboyoun, P.; Pagès, H.; Gentleman, R. ShortRead: a bioconductor package for input, quality assessment and exploration of high-throughput sequence data. *Bioinformatics*. **2009**, 25(19):2607-8. doi: 10.1093/bioinformatics/btp450.

11 Langmead, B.; Salzberg, S.L. Fast gapped-read alignment with Bowtie 2. *Nature Methods.* **2012**, 9: 357-359. doi: 10.1038/nmeth.1923.

12 Dobin, A.; Davis, C.A.; Schlesinger, F.; et al, STAR: ultrafast universal RNA-seq aligner. *Bioinformatics.* **2013**, 29(1): 15-21. doi: 10.1093/bioinformatics/bts635.

13 Li, H.; Handsaker, B.; Wysoker, A.; Fennell, T.; Ruan, J.; Homer, N.; Marth, G.; Abecasis, G.; Durbin, R.; 1000 Genome Project Data Processing Subgroup. The Sequence Alignment/Map format and SAMtools. *Bioinformatics*. **2009**, 25(16):2078-9. doi: 10.1093/bioinformatics/btp352.

14 Liao, Y.; Smyth, G.K.; Shi, W. featureCounts: an efficient general purpose program for assigning sequence reads to genomic features. *Bioinformatics*. **2014,** 30(7):923-30. doi: 10.1093/bioinformatics/btt656.

15 Risso, D.; Schwartz, K.; Sherlock, G.; Dudoit, S. GC-content normalization for RNA-Seq data. *BMC Bioinformatics.* **2011**, 12:480. doi: 10.1186/1471-2105-12-480.
